# Breastfeeding, Bottle Feeding Practices and Malocclusion in the Primary Dentition: A Systematic Review of Cohort Studies

**DOI:** 10.3390/ijerph120303133

**Published:** 2015-03-16

**Authors:** Ana Paula Hermont, Carolina C. Martins, Lívia G. Zina, Sheyla M. Auad, Saul M. Paiva, Isabela A. Pordeus

**Affiliations:** 1 Department of Pediatric Dentistry and Orthodontics, Universidade Federal de Minas Gerais, Belo Horizonte, MG 31270-901, Brazil; E-Mails: anapaulahermont@gmail.com (A.P.H.); smauadtc@googlemail.com (S.M.A.); smpaiva@uol.com.br (S.M.P.); isabela.pordeus@gmail.com (I.A.P.); 2 Department of Public Health, Universidade Federal de Minas Gerais, Belo Horizonte, MG 31270-901, Brazil; E-Mail: liviazina@yahoo.com.br

**Keywords:** malocclusion, breast feeding, bottle feeding, systematic review

## Abstract

The World Health Organization recommends exclusive breast feeding for at least six months. However, there is no scientific evidence of the benefits of breast feeding for oral health in children under primary dentition. This study aimed to search for scientific evidence regarding the following question: is bottle feeding associated with malocclusion in the primary dentition compared to children that are breastfed? An electronic search was performed in seven databases. The systematic review included 10 cohort studies. It was not possible to conduct meta-analysis; therefore a qualitative analysis was assessed. The majority of studies evaluated feeding habits by means of questionnaires and conducted a single examination. Three studies observed that bottle feeding was significantly associated with overjet and posterior crossbite. Studies reported several cut-off times for breastfeeding (varying from 1 month up to 3 years of age) and several types of malocclusion. Controlling for non-nutritive sucking habits was reported for only half of the studies and this may have led to biased results. The scientific evidence could not confirm a specific type of malocclusion associated with the feeding habits or an adequate time of breastfeeding to benefit the children against malocclusion. Further cohort studies are needed to confirm this evidence.

## 1. Introduction

The World Health Organization (WHO) recommends exclusive breastfeeding for the first six months of life to achieve optimal growth, development and health [1]. These recommendations are supported by a systematic review which states the benefits of breastfeeding for six months for minimizing the risk of gastrointestinal infection and growth deficits in young children [2]. Despite the medical benefits of breastfeeding, no systematic review has evaluated the long-term benefits of breastfeeding for oral health, especially related to malocclusion in the primary dentition.

When analyzing malocclusion in the primary dentition the interaction between genetic and environmental factors has to be considered. The most frequently reported environmental factors are changes in feeding habits [3]. Furthermore, it is known that early sucking activity might influence the growth of the craniofacial complex [4]. It is important to keep in mind that malocclusions have negative effects on oral health-related quality of life, predominantly in the dimensions of social and emotional wellbeing [5].

Breastfeeding is reported to be a nutritive sucking habit that protects against malocclusion in the primary dentition [6,7]. Nevertheless, a consensus on this subject has not been established in the literature [8,9]. Some authors report that prolonged breastfeeding decreases the risk of malocclusion [10], others have not found such an association [6,11,12]. Moreover, there is no consensus on the length of time newborn children should be breastfed to protect against malocclusion, as some studies report that six months are sufficient and others report the need for longer periods (6 to 12 months) [13,14,15].

The aim of the present systematic review was to critically evaluate the scientific evidence related to the following clinical question: is bottle feeding associated with malocclusion in the primary dentition when compared to breastfeeding? The PICO question was: (1) Patients: children in the primary dentition phase; (2) Intervention/exposure to risk factor: bottle feeding; (3) Comparison: breastfeeding; and (4) Outcome: malocclusion.

## 2. Materials and Methods

### 2.1. Search Strategy

The following were the inclusion criteria for this systematic review: prospective cohort studies conducted among children ≤seven years of age addressing breastfeeding, bottle feeding and the risk of malocclusion in the primary dentition.

A search was performed in July 2010 and updated in February 2015 by three reviewers (CCM, LGZ, APH) in seven databases: MEDLINE through PubMed (http://www/pubmed.gov), Web of Science (http://www.isiknowledge.com), Cochrane Library (http://www.cochrane.org/index.htm), Clinical Trials (http://controlled-trial.com), UK National Institute for Health and Care Excellence (http://www.nice.org.uk), US National Institutes of Health (http://www.clinicaltrials.gov) and Lilacs and the Brazilian Library of Dentistry (BBO) through the Virtual Health Library (Bireme, Latin America, http://www.bireme.br). No restrictions were placed on language or year of publication. Systematic reviews, theoretical reviews and additional articles of potential relevance were also manually searched. Grey literature was searched from BBO, which retrieved theses and monographies, and from MEDLINE and *The Journal of Breastfeeding Medicine*.

The following strategy was used in MEDLINE, Web of Science and Cochrane: ((malocclusion* OR malocclusion[Mesh] OR dental occlusion[Mesh] OR Maxillofacial Development[Mesh]) AND (bottlefeed* OR bottle feed* OR bottle-feed* OR bottlefed OR bottle fed OR bottle-fed OR “bottle feeding”[Mesh] OR “breast feeding”[Mesh] OR breastfeed* OR breast feed* OR breast-feed* OR breastfed OR breast fed OR breast-fed OR weaning OR “Sucking behavior”[Mesh] OR “Feeding Behavior”[Mesh] OR “risk factors”[Mesh]) NOT (“animals”[Mesh] NOT “humans”[Mesh]). Medline was limited by “humans”. Bireme, Clinical Trials, UK National Institute for Health and Care Excellence and U.S. National Institutes of Health were searched using combined keywords: “bottle feeding”, “breast feeding”, “sucking behavior”, “weaning”, “malocclusion”.

**Figure 1:**
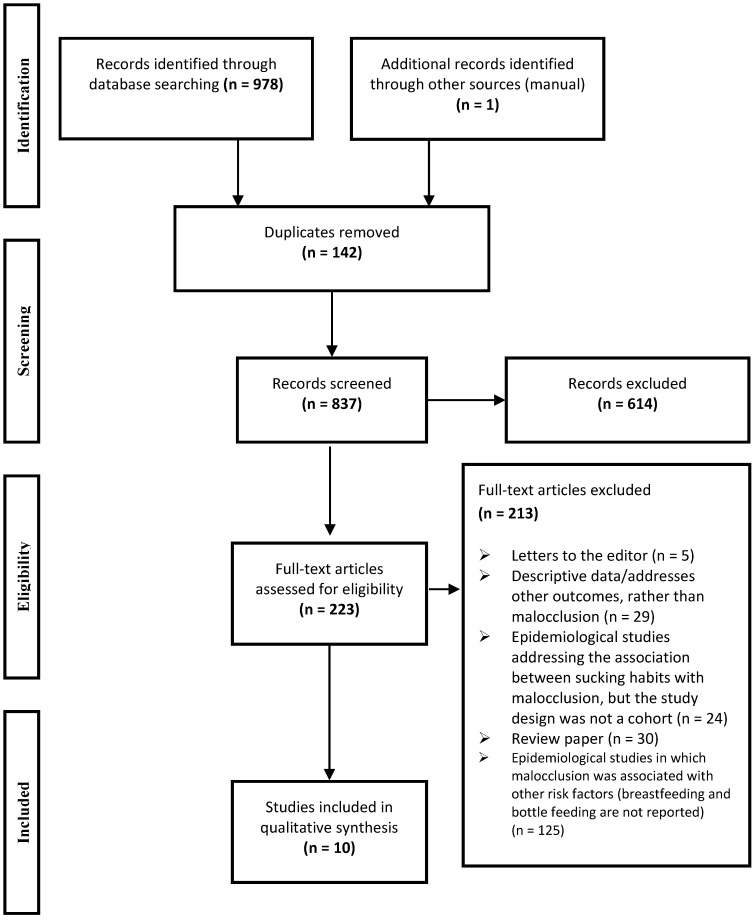
Screening of articles. Four-phase PRISMA flow-diagram for study collection, showing the number of studies identified, screened, eligible, and included in the review and meta-analysis.

The online search retrieved 978 abstracts and titles (Figure 1). The Reference Manager Software^®^ (Reference Manager Version 12.0.3, Thomson Reuters, Paris, France) was used to organize the list of studies. After the removal of duplicate references, 837 studies were initially selected by abstracts and titles by three independent reviewers (CCM, LGZ, APH). The reviewers underwent a calibration process for the selection of studies using the application of inclusion and exclusion criteria. The reviewers thoroughly discussed the criteria, which were applied to 20% of the studies retrieved. This exercise was repeated until adequate agreement based on Cohen’s Kappa coefficient (Kappa: 0.70 to 0.91) was obtained. All studies were then read by the reviewers independently. After applying the criteria, 614 records were excluded by title/abstracts once they did not meet the inclusion criteria.

The following were the exclusion criteria: epidemiological observational studies other than cohorts (e.g., cross-sectional, case-control studies), clinical trials, evaluation of outcomes other than malocclusion (dental trauma, temporomandibular disorders, dental caries, *etc.*); reviews; studies reporting treatment, diagnosis or prevention of malocclusion; risk factors unrelated to feeding habits or infancy; case reports; case series; studies not conducted on humans (laboratorial); studies on food intake; studies addressing the knowledge of parents/dentists regarding oral health; studies conducted with children older than seven years (mixed and permanent dentition); studies reporting only frequency data or surveys; studies on malocclusion conducted on highly selective populations, such as patients with cerebral palsy and Down syndrome, unpublished studies such as abstracts and thesis (for a list of the full texts excluded, see Supplementary material (Table S1).

A total of 223 studies were selected for the analysis of the full texts. Efforts were made to find full texts of abstracts presented in scientific meetings and unpublished theses by personal contact to the authors in order to find in press studies or studies that were under submission.

### 2.2. Data Extraction

Descriptive analysis of the studies was performed (Table 1 and Table 2). Data extraction was conducted by two independent reviewers (APH, CCM). The main outcome was malocclusion (any type of malocclusion), which was considered the endpoint of disease. Malocclusion or types of malocclusion were described according to authors’ definition.

**Table 1:** Quality assessment criteria used for cohort studies through a modified version of Newcastle-Ottawa scale.

**Criteria**	**Cohort Studies and Their Assessment Ratings**									
	Davis & Bell, 1991 [16]	Karjalainen *et al.*, 1999 [17]	Warren & Bishara, 2002 * [12]	Viggiano *et al.*, 2004 [18]	Bishara *et al.*, 2006 * [19]	Vásquez-Nava *et al.*, 2006 [7]	Peres *et al.*, 2007a * [13]	Peres *et al.*, 2007b * [14]	Caramez da Silva *et al.*, 2012 [20]	Moimaz et al., 2014 [21]
**Sample Selection Criteria**										
**(1) Representativeness of the exposed cohort (bottle-fed)** (a) Truly representative sample ★ (b) Somewhat representative of the average community (e.g., hospital) ★ (c) Potential for selection biases or not satisfying requirements in part (a) (d) No description of the derivation of the cohort	b (★)	c	b (★)	a (★)	b (★)	a (★)	a (★)	a (★)	b (★)	b (★)
**(2) Selection of the non-exposed cohort (breastfeeding)** (a) Drawn from the same community as the exposed cohort ★ (b) Drawn from a different source (c) No description of the derivation of the non-exposed cohort	a (★)	a (★)	a (★)	a (★)	a (★)	a (★)	a (★)	a (★)	a (★)	a (★)
**(3) Ascertainment of exposure (bottle feeding)** (a) Data was collected periodically through questionnaires ★ (b) No mention related to the time interval of the feeding habit evaluation/data was collected only once (c) No description	a (★)	a (★)	a (★)	b	a (★)	b	a (★)	a (★)	a (★)	a (★)
**(4) Demonstration that malocclusion was not present at the start of study** (a) yes ★ (b) no description	b	b	b	b	b	b	b	b	b	b
**Comparability of Cohorts on the Basis of the Design or Analysis**										
**(1) Control for confounders** (a) The exposure of interest (malocclusion) is adjusted for the one confounder ★ (b) The exposure of interest (malocclusion) is adjusted for two or more confounders ★★ (c) No description related to the adjustment analysis for confounding factors	c	c	c	a (★)	c	b (★★)	b (★★)	b (★★)	b (★★)	c
**Outcome—Evaluation of Malocclusion**										
**(1) Diagnosis of malocclusion** (a) Clinical examination reporting the use of an index/report of observer agreement—kappa ★ (b) Satisfying requirements in (a) and independent blind assessment ★★ (c) Based on self-reports or not satisfying requirements in part (a, b) (d) No description	c	c	a (★)	c	a (★)	c	a (★)	a (★)	b (★★)	a (★)
**(2) Was follow-up long enough for outcomes (malocclusion) to occur** (a) Yes ★ (b) No	a (★)	a (★)	a (★)	a (★)	a (★)	a (★)	a (★)	a (★)	a (★)	a (★)
**(3) Adequacy of follow up of cohorts** (a) Complete follow up—all subjects accounted for ★ (b) Subjects lost to follow up unlikely to introduce bias, follow up rate ≥ 80% ★ (c) Follow up rate < 80 % or not stated	c	b (★)	c	b (★)	c	c	b (★)	b (★)	c	b (★)
**Summary Score (Stars)**	4/10	4/10	5/10	5/10	5/10	5/10	8/10	8/10	8/10	6/10

★ = one point; ★★ = two points.

**Table 2:** Cohort studies included in the systematic review ordered according to quality score

Authors	Country	Local Setting (Initial and Final Date)	Sample	Children’s Age at Dental Examination	Dental Examination Criteria/Index (Calibration)	Types of Malocclusion Analyzed	Instrument and Time Interval of Feeding Habit Evaluation	Statistics (Adjusted for Confounders)	Outcomes(OR; 95% CI) or (*p*-value)	QualityScore
**Davis & Bell, 1991 [16]**	Canada	National database (Beginning with newborns in 1983 and the children were examined in 1988)	Initial = 670 Final = 108	5 years	Does not report the use of an index/criteria (NR ^†^)	Molar relationships, crossbite, overjet, overbite, crowding, drifting	Questionnaires answered by mothers, monthly, from 1983 to 1988	Chi-square and Kruskal-Wallis (no)	No significant association between malocclusion and feeding method (*p* > 0.05) except for overjet which was associated with exclusive bottle feeding (RR ^‡‡^ = 6.62; *p* = 0.006)	4 (10)
**Karjalainen *et al.*, 1999 [17]**	Finland	Recruited from a prospective baby trial (NR ^†^)	Initial = 179 Final = 148	3 years	Does not report the use of an index/criteria (NR ^†^)	Posterior crossbite, anterior open bite, overjet	Parent interviews recorded 10 times at 1–3 month intervals until the child reached 3 years of age	Chi-square tests and covariance analysis (no)	Children breastfed for 4.7 months had greater frequency of posterior crossbite than children breastfed for 7.6 months (*p* < 0.01). Breastfeeding was not associated with overjet or anterior open bite (*p* > 0.05)	4 (10)
Warren & Bishara, 2002 [12] *	USA	Recruited from hospitals (began with newborns in 1992–1995 and the children were examined at 4.5–5 years of age)	Initial = 700 Final = 372	4.5–5 years	Study models evaluated using Angle classification (NR ^†^)	Primary canine relationship, anterior and posterior crossbite, anterior open bite, overjet, overbite	Questionnaires answered by mothers at 3, 6, 9, 12, 16, 20 and 24 months of age and yearly thereafter	ANOVA(no)	No significant association between malocclusion and duration of breastfeeding (*p* ≥ 0.05)	5 (10)
**Viggiano *et al.*, 2004 [18]**	Italy	Recruited from a school (began with newborns in 1993–1995 and the children were examined in 1998)	Initial = 1130 Final = 1099	3–5 years	Does not report the use of an index/criteria (NR ^†^)	Molar relationships, posterior crossbite, anterior open bite	Structured questionnaire. The data was collected only once	Logistic regression(NNSH ^‡^)	Bottle feeding associated with crossbite (OR: 2.54; 95% CI: 1.66–4.03), but not with open bite (OR: 0.93; 95% CI: 0.65, 1.33) or malocclusion (OR: 1.28; 95% CI: 0.99, 1.66)	5 (10)
Bishara *et al.*, 2006 [19] *	USA	Recruited from hospitals (began with newborns in 1992–1995 and the children were examined at 4.5–5 years of age)	Initial = 547 Final = 372	4.5–5 years	Study models evaluated using Angle classification (NR^†^)	Molar relationships, posterior crossbite, overjet, overbite, anterior open bite	Questionnaires answered by mothers at 3, 6, 9, 12, 16, 20 and 24 months and yearly thereafter	McNemar test (no)	No significant difference between children breastfed for 6–12 months without NNSH and children who were not breastfed but had NNSH <12 months (*p* > 0.05)	5 (10)
**Vázquez-Nava *et al.*, 2006 [7]**	Mexico	NR ^†^	Initial = NR ^†^ Final = 1160	4–5 years	Does not report the use of an index/criteria (NR ^†^)	Anterior open bite, posterior cross bite	Validated questionnaire. The data was collected only once	Qui-square and logistic regression (NNSH ^‡^, allergic rhinitis)	Bottle feeding associated with malocclusion (OR: 1.37; 95% CI: 1.06, 1.78) and crossbite (OR: 1.95; 95% CI: 1.07, 3.54). Bottle feeding was not associated with open bite (OR: 1.27; 95% CI: 0.98, 1.64)	5 (10)
Peres *et al.*, 2007 [13] *	Brazil	Recruited from hospitals (began with newborns in 1993 and the children were examined in 1999)	Initial = 400 Final = 359	6 years	Foster and Hamilton criteria (Kappa ≥ 0.85)	Open bite	Interviews with mothers at 1, 3, 6 and 12 months (1993) and in the child’s fifth year of life	Multivariate analysis (NNSH ^‡^: pacifier/finger sucking, socioeconomic indicators, maternal characteristics)	Open bite was not associated with bottle feeding at 5 years of age in the adjusted analysis (*p* > 0.05). Open bite was associated with breastfeeding <9 months (OR: 2.7; 95% CI: 1.4,6.8, adjusted for dental caries, NNSH^‡^ , maternal schooling and maternal behavioral characteristics)	8 (10)
Peres *et al.*, 2007 [14] *	Brazil	Recruited from hospitals (began with newborns in 1993 and the children were examined in 1999)	Initial = 400 Final = 359	6 years	Foster and Hamilton criteria(Kappa ≥ 0.85)	Anterior open bite, posterior crossbite	Interviews with mothers at 1, 3, 6 and 12 months (1993) and in the child’s fifth year of life	Multivariate analysis, Poisson regression (Time of breastfeeding and NNSH ^‡‡^: pacifier/finger sucking, gender, maternal schooling)	Posterior crossbite was associated with duration of breastfeeding (*p* = 0.036). Posterior crossbite was associated with duration of breastfeeding even after adjustment for the time of NNSH ^‡^ (OR = 7.6; 95% CI: 1.5, 39.5). Anterior open bite was associated with breastfeeding <9 months (*p* = 0.004). After adjustment for the use of pacifier, breastfeeding duration lost significance (OR = 1.2; 95% CI: 0.8, 1.7).	8 (10)
**Caramez da Silva *et al.*, 2012 [20]**	Brazil	Recruited from a hospital (began with newborns in 1993 and the children were examined between 3–5 years-old	Initial = 220 Final = 153	3–5 years	Foster and Hamilton criteria (NR ^†^)	Distocclusion (Class II)	Interview with mothers at 7, 30, 60, 120, 180 days of life and between 3–5 years	Chi-square and Poisson regression (adjusted for duration of pacifier use and bottle-feeding)	Breastfeeding for 12 months or longer protects against canine Class II relation (PR ^††^: 0.44; 95% CI: 0.23, 0.82)	8 (10)
**Moimaz *et al.*, 2014 [21]**	Brazil	Recruited from a program of prenatal care (began with newborns in November 2008 and the children were examined in May 2010)	Initial = 120 Final = 80	30 months	Own criteria(Kappa = 0.92)	Posterior crossbite, anterior crossbite, open bite	Interviews (semi-structured questionnaires) with mothers at 12, 18 and 30 months	Chi-square test and Fisher’s exact test (no)	Posterior crossbite was associated with bottle feeding at 12 and 30 months (*p* = 0.02 and *p* = 0.04, respectively). Overjet > 3mm was associated with breastfeeding at 12 and 18 months (*p* < 0.0001) and at 30 months (*p* = 0.01). Open bite was associated with breastfeeding at 12, 18 and 30 months (*p* < 0.001, *p* = 0.001 and *p* = 0.01, respectively)	6 (10)

* Publications belonging to same epidemiological study reporting different data; **^†^** NR = not reported; **^‡^** NNSH = non-nutritive sucking habits; **^††^** PR = prevalence ratio; **^‡‡^** RR = relative risk.

### 2.3. Assessment of Methodological Quality

The assessment of methodological quality was performed using a modified version of the Newcastle-Ottawa Scale for cohort studies [22]. A system of points (stars) was given to the eligible categories: sequence generation entries, allocation concealment, blinding, incomplete outcome data, and sample losses. The scale ranged from 0 (lowest grade) to 10 (highest grade). Studies with scores above the median were classified as high quality studies. Each cohort could be awarded a maximum of one point (star) for each numbered item, except for the “Comparability” criteria and ‘Evaluation of malocclusion’, in which a maximum of two stars could be scored (Table 1).

### 2.4. Data Synthesis

There was substantial clinical heterogeneity among studies, due to the different types of malocclusion described in the studies and the different cut-off times used to evaluate feeding habits. As data was not similar enough to be combined it was not possible to group data to conduct meta-analysis. For this reason, a qualitative analysis was conducted. Publication bias was not quantitatively evaluated as there were not enough studies to be grouped in a funnel plot [23,24].

## 3. Results

### 3.1. Characteristics of Studies

Among the 223 studies selected for the full-text analysis, 10 cohort studies were included in the present systematic review (Figure 1). No clinical trials were found. Four large epidemiological studies were identified as having multiple publications reporting different data: two American cohort studies [12,19] and two Brazilian cohort studies [13,14]. There were cohorts from Canada (16) [16], Finland [17], Italy [18], Mexico [7], USA [12,19] and Brazil [13,14,20,21] (Table 2). In all studies the feeding habits were evaluated longitudinally from birth and at specific time intervals until the end of the study, except for two studies in which the data was collected only once [7,18]. All studies performed only one dental examination; the outcome (malocclusion) was evaluated at ages between 3 to 6 years.

### 3.2. Qualitative Analysis

#### 3.2.1. Type of Feeding Habit and Type of Malocclusion

The findings on associations between types of feeding habits and types of malocclusion were divergent. A study conducted between 1983 and 1988 observed that the feeding method was not associated with malocclusion (*p* > 0.05), except for overjet which was associated with exclusive bottle feeding (*p* = 0.006) [16]. Other authors found that bottle feeding was significantly associated with posterior crossbite (OR = 1.95) [7], (OR = 2.54) [18] and (*p* < 0.001) [21]. Confounders were hardly reported and adjustments for non-nutritive sucking habits were performed in performed in only half of the studies [7,13,14,18,20].

#### 3.2.2. Duration of Breastfeeding and Malocclusion

Despite the lack of evidence, it seems that longer breastfeeding duration favors normal occlusion [13,14,17,20]. Studies found that longer periods of breastfeeding decreased the occurrence of posterior crossbite and open bite [13,14,17] and breastfeeding for more than nine months protected against open bite compared to children breastfed for less than nine months [13]. Moreover, it was observed that breastfeeding for 12 months or longer protected against malocclusion (distocclusion). Nevertheless, one study observed that the duration of breastfeeding was not associated with malocclusion [12].

### 3.3. Quality Assessment

The methodological quality evaluated using the Newcastle-Ottawa scale ranged from a score of 3 to 8 (maximum: 10) (Table 1 and Table 2). The main shortcomings were related to the data collection process. Oral examinations were conducted only once and the children’s age at dental examinations varied between 3 to 6 years. No study performed a baseline oral examination to ensure that the volunteers were free of malocclusion at the beginning of the study. Although the subjects were recruited at birth, the lack of follow up throughout the study period did not allow the determination of whether the malocclusion underwent changes over the years or the age that malocclusion began. Another shortcoming was the absence of controlling for confounders in the statistical analysis. Only five studies controlled for non-nutritive sucking habits [7,13,14,18,20]. The adequacy of follow up of cohorts constituted another limitation; only five studies reported a follow up rate ≥80% [13,14,17,18,21].

## 4. Discussion

### 4.1. Assessment of Bias

In the present systematic review, no bias occurred due to language or year of publication. All cohorts included in this systematic review were published in English [7,12,13,14,16,17,18,19,20,21]. The search involved papers published between 1991 [16] and 2014 [21]. Efforts were made to find breastfeeding/bottle feeding habits in papers as a confounder, but not as the main subject. The full text of 223 studies was analyzed.

### 4.2. Assessment of Quality

Substantial methodological and clinical heterogeneity was found among the studies. The major shortcomings were the failure to report that the outcome of interest was not present at the start of the study (absence of an initial dental examination), the non-adequacy of follow-up rates (the time intervals of feeding habits evaluation varied a lot) and the failure to control for confounders, especially non-nutritive sucking habits.

Controlling for confounders can be adjusted by multivariate analysis or by excluding children with non-nutritive sucking habits from the analysis. Pacifier use or thumb sucking are confounder variables, since is not possible to determine whether malocclusion was caused by bottle feeding or pacifier/thumb sucking. Another problem was the sample losses during the study. Although sample losses can be expected in cohort studies, only half of the studies reported follow up rates >80% [13,14,17,18,21].

Details on the blinding process related to the dental examinations were fairly reported. If the same examiner evaluates the malocclusion and the data about feeding habits, the diagnosis of malocclusion can be biased. Only one study reported blinding procedures [20].

Information bias is another shortcoming. Mothers and proxy respondents can be mistaken about feeding habits or can answer the option that they feel is correct in order to please the interviewer. Although the majority of studies evaluated feeding habits longitudinally, in all cohorts malocclusion was evaluated only once, at the end of the study. The follow up of dental condition is important since it can provide information related to the age that malocclusion started or if the malocclusion self-corrected at the time of the final examination.

### 4.3. Strength of Evidence

The evidence did not confirm a specific type of malocclusion related to bottle feeding, as the studies reported divergent findings. Several types of malocclusion were evaluated and punctual evidences were found associating bottle feeding with overjet [16] and posterior crossbite [7,18,21]. Nevertheless, it is not possible to confirm if overjet and crossbite are significantly associated with bottle feeding, since there were only three studies addressing this data and they did not have a good methodological quality (Table 1). Furthermore, there was a lack of reporting of calibration exercises for clinical data collection and blind assessments.

In general, it seems that prolonged breastfeeding can protect against malocclusion or favor normal occlusion. Malocclusion herein is considered to be “any type of malocclusion”, since many studies used this classification. However, several types of malocclusion were analyzed, such as posterior crossbite, open bite and distocclusion [13,14,17,21]. Cut-off times for breastfeeding varied a lot.

The clinical heterogeneity among studies disrupts the strength of evidence. Moreover, the absence of controlling for confounders, as pacifier or digit sucking habit, which can also be associated with malocclusion should be pointed out. Exclusion of children that had non-nutritive sucking habits from the sample could be a good strategy in studies evaluating this subject. Another option is to consider non-nutritive sucking habits in a multivariate model analysis.

When analyzing causality it is important to consider Hill's criteria of causation which consists of nine items: strength of association, consistency, specificity, temporality, dose response, experimental evidence, biological plausibility, coherence, and analogy [25]. Hill’s criteria of causation were applied to the possible causal relationship between feeding habits and malocclusion (any type of malocclusion).

There is no experimental evidence related to this issue; no clinical trials were found probably due to ethical issues, therefore causality between malocclusion and feeding habits cannot be confirmed yet (Table 3). Despite the weak evidence regarding the association between bottle feeding for more than one year and malocclusion in the primary dentition, it seems prudent to interrupt this habit as soon as possible until further evidence is obtained.

**Table 3:** Hill’s criteria of causation applied to malocclusion and its association with breastfeeding and bottle feeding.

Criteria	Definitions	Causal Relationship between Bottle Feeding/Breastfeeding and Malocclusion
**Strength**	How strong is the association between the cause and the effect?	It seems that prolonged breastfeeding can protect against malocclusion or favour normal occlusion The evidence related to the association between malocclusion and bottle feeding is weak; the studies reported divergent findings
**Consistency**	The association is consistent when results are replicated in studies in different settings using different methods	The studies were conducted among children from different countries and different methods were applied
**Specificity**	The cause leads to a single effect. The more specific an association between a factor and an effect is, the bigger the probability of a causal relationship	Despite the lack of evidence, the majority of studies linked bottle feeding to some type of malocclusion
**Temporality**	The cause precedes the effect	It seems that the cause (bottle feeding) occurs before the effect (malocclusion), but the studies did not conduct an initial dental examination in order to prove that the outcome of interest was not present at the start of study
**Biological gradient**	Also known as dose response. Greater exposure should generally lead to greater risk of the disease/ effect	Despite the lack of evidence, it seems that longer breastfeeding duration favours normal occlusion It seems prudent to interrupt bottle feeding as soon as possible until further evidence is obtained
**Plausibility**	The effect must have biologic plausibility	The habit of sucking an object such as a bottle which is related to feeding habits, involves patterns of muscle contraction in the orofacial region and may cause malocclusion
**Coherence**	Coherence between epidemiological and laboratory findings increases the likelihood of an effect	There were no studies indicating a credible level of coherence
**Experimental evidence**	Experimental or semi-experimental evidence exists to support the causation hypothesis	There were no studies demonstrating malocclusion in the animal model
**Analogy**	The effect of similar factors may be considered	There were no studies demonstrating malocclusion in the animal model

The American Academy of Pediatrics recommends parents to encourage their infants aged one year to be weaned using cups instead of a bottle in order to avoid early childhood caries [26]. WHO recommends that children should be breastfed exclusively for at least six months [1,2], as this length of time benefits systemic health.

The cohort study design has higher levels of evidence [27] and is less prone to methodological bias and recall bias regarding feeding habits. However, a small number of cohort studies were included in the present study. Besides evaluating non-nutritive sucking habits and malocclusion, many papers had to be excluded since there was no mention of breastfeeding/bottle feeding. The absence of reporting findings that did not achieve statistical significance, such as breastfeeding/ bottle feeding, may have led to bias.

Despite the higher level of evidence of cohort studies, methodological designs with lower levels of evidence should not be ignored. Thus, caution should be exercised when interpreting the results [27]. There is a lack of cohort studies evaluating malocclusion and breastfeeding in the literature. Nevertheless, the evidence reported herein is currently the most reliable until new observational cohort studies are conducted.

## 5. Conclusions

The scientific evidence could not confirm the types of malocclusion associated with bottle feeding or a proper period for breast feeding in order to protect against malocclusion. Until further studies are conducted to confirm the evidence related to the association between bottle feeding and malocclusion, exclusive breastfeeding for at least six months of age is still the best recommendation to benefit children regarding their systemic health. The present findings reveal substantial heterogeneity regarding feeding habits and types of malocclusion. Further observational cohort studies with longitudinal data on feeding habits and malocclusion are needed to confirm this evidence.

## Supplementary Files

Supplementary File 1
