# Supplementary material for: Breastfeeding, Bottle Feeding Practices and Malocclusion in the Primary Dentition: A Systematic Review of Cohort Studies
Source: Int J Environ Res Public Health. 2015 Mar 16;12(3):3133–51. doi: 10.3390/ijerph120303133 (PMC4377956; doi:10.3390/ijerph120303133)
Supplement: Supplementary File 1 [file ijerph-12-03133-s001.pdf]

Supplementary Information

Breastfeeding, Bottle Feeding Practices and Malocclusion in the Primary Dentition: A Systematic Review of Cohort Studies

Table S1. List of titles selected for full text analysis and the reasons for exclusion.

References	Classification
Adair, S.M.; *et al*., 1992 [1]	Not selected—The study correlates malocclusion with the use of orthodontic pacifiers. No feeding habits are reported.
Adamiak, E.; 1981 [2]	Not selected—The study does not associate malocclusion to bottle or breast feeding.
Ahangar Atashi, M.H.; *et al*., 2013 [3]	Not selected—The study does not associate malocclusion to bottle or breast feeding.
Ahlgren, J.; 1995 [4]	Not selected—The study evaluated lip and cheek activity; no risk factors or malocclusion were evaluated.
Al-Bajjali, T.T.; *et al*., 2014 [5]	Not selected—The study does not associate malocclusion to bottle or breast feeding.
Alexander, L.G.; 1981 [6]	Not selected—The study does not associate malocclusion to bottle or breast feeding.
Alexander, S.; *et al*., 1997 [7]	Not selected—The study reports malocclusion and periodontal status. No bottle or breast feeding are reported.
Almeida, S.P.T.M.A.; *et al*., 2005 [8]	Not selected—Review.
Antonini, A.; *et al*., 2005 [9]	Not selected—The study associates malocclusion to skeletal characteristics based on cephalograms. No breast feeding or bottle feeding is reported.
Aravena, A.P.; *et al*., 1998 [10]	Not selected—The study is a review.
Aznar, T., *et al*.; 2006 [11]	Not selected—Cross-sectional study.
Backlund, E.; *et al*., 1963 [12]	Not selected—The study reports breathing.
Baragona, P.M.; *et al*., 1991 [13]	Not selected—Review.
Baume, L.J.; 1973 [14]	Not selected—The study reports frequency of oral problems in French Polynesian population; no risk factors are reported.
Baume, L.J.; 1974 [15]	Not selected—Study describes the prevalence of malocclusion and dental eruption pattern in Polynesian population. Risk factors are not reported.
Bertoldi, P.M.; *et al*., 2005 [16]	Not selected—The study reports the effect of counseling on interruption of oral habits.
Bertrand, F.R.; 1968 [17]	Not selected—The study does not report epidemiological and quantitative data on breast feeding.
Bigenzahn, W.; *et al*., 1992 [18]	Not selected—The study describes the effect of myofunctional therapy on speech and disorders of articulation.
Boni, R.C.; *et al*., 1997 [19]	Not selected—The study reports the effects of counseling on malocclusion.
Borghoff, M.J.; *et al*., 2005 [20]	Not selected—The study is conducted with children aged 7 to 11 years and dental examination was not performed.
Bowden, B.D.; 1966 [21]	Not selected—The study does not report breast feeding or bottle feeding; only non-nutritive sucking habits.

Table S1. *Cont.*

**References**	**Classification**
Caglar, E.; *et al*., 2005 [22]	Not selected—The study reports prevalence data.
Callaghan, A.; *et al*., 2005 [23]	Not selected—Pacifier use is the main outcome.
Calo, F.A.; 1968 [24]	Not selected—Review
Carrascoza, K.; *et al*., 2006 [25]	Not selected—Comparison group to bottle feeding was children that used glass (instead of bottle). Data of malocclusion are not described in the paper.
Carrero H.B.; *et al*., 1988 [26]	Not selected—Review.
Castellani, G.; *et al*., 1987 [27]	Not selected—The study does not report breast feeding or bottle feeding; only non-nutritive sucking habits.
Castelo, P.M., *et al*.; 2010 [28]	Not selected—Cross-sectional study.
Castillo, B.; 2008 [29]	Not selected—Review.
Castro, L.A.; 2002 [30]	Not selected—The study evaluates the primary dentition and its development.
Charchut, S.W.; *et al*., 2003 [31]	Not selected—Cross-sectional study
Chevitarese, A.B.; *et al*., 2002 [32]	Not selected—The study does not associate malocclusion to bottle or breast feeding; only oral habits are reported.
Costantino, A.; 1986 [33]	Not selected—The study has no statistical data.
Courson, F.; 2006 [34]	Not selected—Review.
Cozza, P.; *et al*., 2005 [35]	Not selected—The study does not report breast feeding or bottle feeding.
Degano, M.P.; Degano, R.A.; 1993 [36]	Not selected—Review.
Del Valle, L.M.L.; *et al*., 2006 [37]	Not selected—Cross-sectional study.
Dimberg, L.; *et al*., 2010 [38]	Not selected—The study does not report breast feeding or bottle feeding; the study evaluated non-nutritive sucking habits, breathing pattern and snoring.
Dimberg, L.; *et al*., 2013 [39]	Not selected—The study does not report breastfeeding.
Diouf, J.S.; *et al*., 2010 [40]	Not selected—Cross-sectional study.
Djaha, K.; *et al*., 1986 [41]	Not selected—The study does not associate malocclusion to breast feeding or bottle feeding.
Dolci, G.S., *et al*., 2001 [42]	Not selected – The study does not report breast feeding or bottle feeding; only associated non-nutritive sucking habits.
Dos Santos, R.R.; *et al*. 2012 [43]	Not selected—Cross-sectional study.
Duncan, K.; *et al*., 2008 [44]	Not selected—The study does not report breast feeding or bottle feeding; only non-nutritive sucking habits.
Joanna Briggs Institute. 2006 [45]	Not selected—Review.
Emmerich, A.; *et al*., 2004 [46]	Not selected—The study does not report breast feeding or bottle feeding; only non-nutritive sucking habits and oronasopharyngeal alterations.
Eskes, P.W.; 1992 [47]	Not selected—Letter to the Editor.
Fabac, E., *et al*.; 1992 [48]	Not selected—Cross-sectional study.
Farsi, N.M.; *et al*., 1997 [49]	Not selected—The study does not correlate breast feeding or bottle feeding to malocclusion; it is related only to non-nutritive sucking habits.
Ferreira, S.H.; *et al*., 2001 [50]	Not selected—The study describes prevalence data of open bite; breast feeding or bottle feeding were not evaluated.
Ferrer, L.; *et al*., 2006 [51]	Not selected—Cross-sectional study.

Table S1. *Cont.*

**References**	**Classification**
Ferro, A.; *et al*., 1979 [52]	Not selected—The study does not associate malocclusion to bottle or breast feeding.
Fote, F.D.; *et al*., 2000 [53]	Not selected—The study does not report outcomes of malocclusion, breast feeding or bottle feeding.
Fourquet, L.; *et al*., 2014 [54]	Not selected—The study does not associate malocclusion to bottle or breast feeding.
Frazao, P.; *et al*., 2006 [55]	Not selected—The study analyses epidemiological survey of the Brazilian Health Authority. No risk factors are reported.
Furtado, A.N.M.; *et al*., 2007 [56]	Not selected—Cross-sectional study.
Galan-Gonzalez, A.F.; *et al*., 2014 [57]	Not selected—Cross-sectional study.<br>The study addresses the influence of breastfeeding and bottle feeding on development of the dental arches and occlusion among children aged from 3 to 6 years.
Gallarreta, F.W.M.; *et al*., 2004 [58]	Not selected—The study associates breast feeding to sucking habits.
Gandini, P.; *et al*., 1989 [59]	Not selected—The study describes only frequency data.
Gariner, D.; 1970 [60]	Not selected—The study is a review.
Garliner, D.; 1984 [61]	Not selected—The study does not associate malocclusion to bottle or breast feeding.
Gedicke, K.; 1961 [62]	Not selected—The study does not associate malocclusion to breast feeding or bottle feeding.
Gimenez, C.M.M.; *et al*., 2001 [63]	Not selected—Abstract.
Gimenez, C.M.M.; *et al*., 2008 [64]	Not selected—The study includes children with special needs what can be a confounder, since this population are more prompt to malocclusions. Data extraction was not possible.
Gimenez, C.M.M.; *et al*., 2008 [65]	Not selected—The study describes *p*-value of the statistics between malocclusion and feeding habits, but it was not possible to extract data.
Gois, E.G.; *et al*., 2008 [66]	Not selected—The study does not report breast feeding or bottle feeding. It associates malocclusion to other risk factors.
Grabowski, R.; *et al*., 2007 [67]	Not selected—The study does not report breast feeding or bottle feeding.
Gudin, R.G.; *et al*., 1993 [68]	Not selected—Review.
Gyorgy, I.; 1970 [69]	Not selected—The study does not associate malocclusion to bottle or breast feeding.
Hannuksela, A.; *et al*., 1987 [70]	Not selected—The study does not report breast feeding or bottle feeding, only associates malocclusion to other risk factors.
Harrel, S.K.; 2005 [71]	Not selected—Letters to the Editor.
Hashida, S.; *et al*., 1985 [72]	Not selected—The study does not associate malocclusion to breast feeding or bottle feeding.
Hebling, S.R.; *et al*., 2008 [73]	Not selected—Cross-sectional study.
Heimer, M.V.; *et al*., 2010 [74]	Not selected—The study does not report breast feeding or bottle feeding; only non-nutritive sucking habits and growth pattern are reported.
Heimer, M.V.; *et al*., 2008 [75]	Not selected—The study associates malocclusion to non-nutritive sucking habits and facial morphology; no breast feeding or bottle feeding is reported.

Table S1. *Cont.*

**References**	**Classification**
Helle, A.; *et al*., 1974 [76]	Not selected—The study does not report breast feeding or bottle feeding; only non-nutritive sucking habits.
Holberg, C.; *et al*., 2013 [77]	Not selected—The study does not associate malocclusion to bottle or breast feeding.
Homan, B.T.; *et al*., 1973 [78]	Not Selected—The study is a survey of oral disease in Aborigines population. No risk factors are reported.
Ito, G.; 1984 [79]	Not selected—The study describes skeletal traits.
Iwashima, Y.; *et al*., 2014 [80]	Not selected—The study does not associate malocclusion to bottle or breast feeding.
Jabbar, N.S.; *et al*., 2011 [81]	Not selected—Cross-sectional study.
Jain, S., *et al*.; 2014 [82]	Not selected—The study does not associate malocclusion to bottle or breast feeding.
Katz, C.R.; *et al*., 2004 [83]	Not selected—The study does not report breast feeding or bottle feeding, but other risk factors.
Katz, C.R.; *et al*., 2005 [84]	Not selected—The study does not report breast feeding or bottle feeding; only non-nutritive sucking habits.
Katz, C.R.T.; 2003 [85]	Not selected—The study is a thesis and it was published in scientific periodicals. Papers published were read and not selected. The study is about non-nutritive sucking habits.
Kieser, J.J. ; 2002 [86]	Not selected—Letter to the Editor.
Kimmel, K.; 1990 [87]	Not selected—The study does not associate malocclusion to breast feeding or bottle feeding.
Kimmel, K.; 1980 [88]	Not selected—Review.
Kisling, E.; *et al*., 1976 [89]	Not selected—The study is a survey of patterns of occlusion in 3 year-old children. Oral habits are seldom reported as non-nutritive sucking habits associated to malocclusion.
Kobayashi, H.M.; *et al*., 2010 [90]	Not selected—Cross-sectional study.
Kobayashi, H.M.; *et al*., 2008 [91]	Not selected—The study reports sucking habits and malocclusion.
Koev, Z.H.; 1969 [92]	Not selected—Review.
Kuijpers-Jagtman, A.M.; 1989 [93]	Not selected—Review.
Labbok, M.H.;*et al*., 1987 [94]	Not selected—The study includes children aged 3 to 18 years and it was not possible to split children by age or collect data of only primary dentition.
Larsson, E.; *et al*., 1971 [95]	Not selected—Non-nutritive sucking habits is the main outcome.
Larsson, E.; 1994 [96]	Not selected—Review.
Larsson, E.; 1975 [97]	Not selected—Non-nutritive sucking habits are the main outcome.
Larsson, E.; 1971 [98]	Not selected—Non-nutritive sucking habits are the main outcome.
Larsson, E.; 1972 [99]	Not selected—Non-nutritive sucking habits are the main outcome.
Larsson E.; 1986 [100]	Not selected—The study does not report breast feeding or bottle feeding; only non-nutritive sucking habits.
Larsson, E.; 1998 [101]	Not selected—The study does not evaluate malocclusion; only feeding practices.

Table S1. *Cont.*

References	Classification
Larsson, E.; 2001 [102]	Not selected—The study does not associate breast feeding or bottle feeding to malocclusion. Breast feeding is only associated to sucking habits.
Larsson, E.; 1986 [103]	Not selected—Review.
Larsson, E.F.; *et al*., 1985 [104]	Not selected—The study describes prevalence of sucking habits, and does not report association of breast feeding or bottle feeding with malocclusion.
Legovic, M.; *et al*., 1991 [105]	Not selected—Cross-sectional study.
Leighton, B.C.; 1966 [106]	Not selected—Risk factors are seldom reported as non-nutritive sucking habits. Breast feeding or bottle feeding are not reported.
Leite-Cavalcanti, A.; *et al*., 2007 [107]	Not selected—Cross-sectional study.
Linder, A.; *et al*., 1989 [108]	Not selected—The study does not report breast feeding or bottle feeding; only non-nutritive sucking habits.
Luft, R.; 1977 [109]	Not selected—The study does not associate malocclusion to breast feeding or bottle feeding.
Luz, C.L.; *et al*., 2006 [110]	Not selected—Cross-sectional study.
Macena, M.C.; *et al*., 2009 [111]	Not selected—The study associate malocclusion to socioeconomic factors and to non-nutritive sucking habits; no breast feeding or bottle feeding is reported.
Maciel, C.T.; *et al*., 2005 [112]	Not selected—The study does not associate feeding habits to malocclusion.
Maggioni, A.; 1984 [113]	Not selected—Review
Mahalski, P.A.; *et al*., 1992 [114]	Not selected—The study is about digit sucking habits associated to behavior problems; no breast feeding or bottle feeding is reported.
Malagola, C.; *et al*., 1986 [115]	Not selected—The study does not associate malocclusion to breast feeding or bottle feeding.
Martins, R.J.; *et al*., 2003 [116]	Not selected—The study does not report breast feeding or bottle feeding; only associated non-nutritive sucking habits.
Martori, E., *et al*.; 2014 [117]	Not selected—The study does not associate malocclusion to bottle or breast feeding.
Masaracchia, R.; 2009 [118]	Not selected—Review.
Massler, M.; 1983 [119]	Not selected—The study does not associate malocclusion to breast feeding or bottle feeding.
Melink, S.; *et al*., 2010 [120]	Not selected—Case-control study.
Melsen, B.; *et al*., 1979 [121]	Not selected—The study does not report breast feeding or bottle feeding; it associates malocclusion to non-nutritive sucking habits and swallowing pattern.
Mendes, A.C.R.; *et al*., 2003 [122]	Not selected—Abstract.
Mendes, A.C.R.; *et al*., 2008 [123]	Not selected—The study does not report outcome of breast feeding. Breast feeding is only associated to non-nutritive sucking habits.
Meyers, A.; *et al*., 1998 [124]	Not selected—The study comprises children aged 10 to 12 years (mixed dentition) and malocclusion is evaluated as parents' report of need of orthodontic treatment.
Michelotti, A.; *et al*., 2007 [125]	Not selected—The study associates malocclusion to leg length inequality in adolescents; no breast feeding or bottle feeding is reported.

Table S1. *Cont.*

**References**	**Classification**
Milicic, A.; 1972 [126]	Not selected—The study reports prevention and orthodontics.
Montaldo, L.; *et al*., 2011 [127]	No selected—The study only associates feeding habits to non-nutritive sucking habits.
Moraes, E.S.; *et al*., 2001 [128]	Not selected—The study does not report breast feeding or bottle feeding; only associated non-nutritive sucking habits.
Moss, J.P.; *et al*., 1968 [129]	Not selected—The study is conducted with children aged 12 years.
Motokawa, M.; *et al*., 2013 [130]	Not selected—The study does not associate malocclusion to bottle or breast feeding.
Mustafa, M.; *et al*., 2014 [131]	Not selected—The study does not associate malocclusion to bottle or breast feeding.
Myllarniemi, S.; 1973 [132]	Not selected—The study does not report breast feeding or bottle feeding; only non-nutritive sucking habits
Narbutyte, I.; *et al*., 2013 [133]	Not selected—Review.
Neiva, F.C.; *et al*., 2003 [134]	Not selected—Review.
Nowak, A.J.; 1991 [135]	Not selected—Report.
Nystrom, M.; 1981 [136]	Not selected—The study reports changes of malocclusion during years; no risk factors are reported.
Obraztsov, I.; 1991 [137].	Not selected—The study does not associate malocclusion to breast feeding or bottle feeding.
Ogaard, B.; *et al*., 1994 [138]	Not selected—Cross-sectional study.
Oikawa, K.; 1984 [139]	Not selected—Review.
Oliveira, A.B.; *et al*., 2006 [140]	Not selected—Cross-sectional study.
Mew, J.; 2002 [141]	Not selected—Treatment study.
Onyeaso, C.O.; *et al*., 2008 [142]	Not selected—The study does not report breast feeding or bottle feeding; only non-nutritive sucking habits.
Onyeaso, C.O.; *et al*., 2008 [143]	Not selected—The study reports non-nutritive sucking habits and malocclusion.
Onyeaso, C.O.; 2004 [144]	Not selected—The study does not associate malocclusion to breast feeding or bottle feeding.
Ovsenik, M., *et al*.; 2007 [145]	Not selected—The study longitudinally follows up children to evaluate changes of malocclusion pattern during years. Bottle feeding of children with malocclusion is reported along the years, but there is no information of bottle feeding in children without malocclusion. OR is not reported and it was not possible to obtain this data from the paper.
Ovsenik, M.; 2009 [146]	Not selected—Breast feeding is not reported. Bottle feeding is reported along years, and there is no frequency data or that could be extracted.
Page, D.C.; 2001 [147]	Not selected—Review.
Pal, A.; 1976 [148]	Not selected—The study does not associate malocclusion to bottle or breast feeding.
Paunio, P.; *et al*., 1993 [149]	Not selected—Breast feeding and bottle feeding are not associated to malocclusion, are only associated to sucking habits.
Paunio, P.; *et al*., 1993 [150]	Not selected—Non-nutritive sucking habits are the main outcome.
Peres, K.G.; *et al*., 2006 [151]	Not selected—Abstract.

Table S1. *Cont.*

**References**	**Classification**
Peters, S.; 1988 [152]	Not selected—Review.
Piras, V.; *et al*., 2003 [153]	Not selected—The study is comprised of a selected group of patients with beta-thalassemia and its association with malocclusion.
Popovich, F.; 1966 [154]	Not selected—The study does not associate malocclusion to bottle or breast feeding.
Primozic, J.; *et al*., 2014 [155]	Not selected—The study does not associate malocclusion to bottle or breast feeding.
Radovic, K., *et al*.; 2014 [156]	Not selected—The study does not associate malocclusion to bottle or breast feeding.
Rakosi, T.; 1969 [157]	Not selected—The study reports prevention methods.
Rakosi, T.; 1972 [158]	Not selected—Review.
Raymond, J.L.; *et al*., 2006 [159]	Not selected—Review.
Ribeiro, H.C., Jr.; 2005 [160]	Not selected—It is a case-control study.
Robke, F.J.; 2008 [161]	Not selected—The stud correlates nursing bottle to its consequences such as nursing bottle caries and malocclusion. Types of malocclusion reported are crowding and premature loss of teeth and space in consequence to nursing bottle caries; not malocclusion caused by bottle feeding.
Roedig, J.J.; *et al*., 2014 [162]	Not selected—The study does not associate malocclusion to bottle or breast feeding.
Romero, C.C.; *et al*., 2011 [163]	Not selected—Cross-sectional study.
Saenz, R.B.; 2000 [164]	Not selected—Letters to the Editor.
Salma, S.; 2000 [165]	Not selected—Review.
Sanchez-Molins, M.; *et al*., 2010 [166]	Not selected—The study is comprised of children aged 6 to 11 years under mixed dentition.
Sanger, R.G.; *et al*., 1982 [167]	Not selected—The study is not original paper.
Scavone, H.; *et al*., 2008 [168]	Not selected—The study does not associate breast feeding or bottle feeding to malocclusion; breast feeding is associated to non-nutritive sucking habits.
Scavone, H., *et al*.; 2007 [169]	Not selected—The study does not report breast feeding or bottle feeding; only non-nutritive sucking habits.
Schneider, H.G.; *et al*., 1989 [170]	Not selected—The study does not report malocclusion as main outcome.
Shekar, B.R.; *et al*., 2013 [171]	Not selected—The study does not associate malocclusion to bottle or breast feeding.
Shendurnikar, N.; 1993 [172]	Not selected—Review.
Shetty, S.R.; *et al*. 1998 [173]	Not selected—Is a prevalence study.
Silva, L.P.M.; *et al*., 2005 [174]	Not selected—Cross-sectional study.
Simpson, W.J.; *et al*., 1976 [175]	Not selected—There is no statistical data, only frequency is reported. It was not possible to extract data.
Simpson, W.J.; *et al*., 1976 [176]	Not selected—There is no statistical data, only frequency is reported. It was not possible to extract data.
Solano, R.E.; *et al*., 1988 [177]	Not selected—The study does not associate malocclusion to bottle or breast feeding.

Table S1. *Cont.*

**References**	**Classification**
Sorensen, H.B.; *et al*., 2009 [178]	Not selected—The study associates ectopic canines to their location in the maxilla; no malocclusion or risk factors are related.
Sousa, J.M.; *et al*., 2010 [179]	Not selected—The study does not associate breast feeding or bottle feeding to malloclusion; only prevalence data is reported.
Souza Júnior, M.A.; 1998 [180]	Not selected—The published study was identified and selected. The thesis was excluded in order to avoid duplicate data.
Soviero, V.L.M.; 1999 [181]	Not found.
Stahl, F., *et al*.; 2003 [182]	Not selected—The study does not associate malocclusion to bottle or breast feeding.
Starr, N.B.; *et al*., 1999 [183]	Not selected—The study does not report breast feeding or bottle feeding.
Stecksen-Blicks, C.; *et al*., 1995 [184]	Not selected—The study reports frequency of dental caries, dental trauma and malocclusion in Swedish children. There is no association with risk factors.
Stirling, D.L.; 1994 [185]	Not selected—Review.
Taatz, H.; 1978 [186]	Not selected—Review.
Takanashi, N.; *et al*., 1989 [187]	Not selected—The study reports prevalence data.
Thomaz, E.B.; *et al*., 2013 [188]	Not selected—The study includes children with permanent dentition with 12–15 years-old, and does not associate malocclusion to feeding habits.
Thomaz, E.B.; *et al*., 2012 [189]	Not selected—The study was conducted with adolescents aged 12–15 years. No primary dentition.
Thomaz, E.B.A.F.; *et al*., 2005 [190]	Not selected—The study does not report breast feeding or bottle feeding as risk factors.
Thomazine, G.D.P.A.; *et al*., 2000 [191]	Not selected—The study describes prevalence data of open bite; breast feeding or bottle feeding were not evaluated.
Tollaro, I.; *et al*., 1968 [192]	Not selected—The study reports sucking habits.
Tomita, L.M.; *et al*., 2004 [193]	Not selected—The study does not associate feeding habits and malocclusion.
Tomita, N.E.; *et al*., 2000 [194]	Not selected—The study does not report breast feeding or bottle feeding; only non-nutritive sucking habits.
Tsai, H.H.; *et al*., 2004 [195]	Not selected—The study does not associate malocclusion to bottle or breast feeding.
Tschill, P.; *et al*., 1997 [196]	Not selected—The study reports prevalence of malocclusion in children; it does not report risk factors.
Turgeon-O'Brien, H.; *et al*., 1996 [197]	Not selected—Review.
Urzal, V.; *et al*., 2013 [198]	Not selected—The study does not report breastfeeding.
Usadel, W.; 1967 [199]	Not selected—The study is comprised of a selected group of patients from clinical practice.
Usadel, W.; 1970 [200]	Not selected—The study does not have sufficient quantitative data for data's extraction.
Vasconcelos, F.M.; *et al*., 2011 [201]	Not selected—Cross-sectional study.
Verrastro, A.P.; *et al*., 2006 [202]	Not selected—The study associates malocclusion to orofacial myofunctional characteristics. Breast feeding or bottle feeding are not reported.
Verrastro, A.P.; 2008 [203]	Not selected—Cross-sectional study.

Table S1. *Cont.*

References	Classification
Vieira-Andrade, R.G.; *et al.*, 2014 [204]	Not selected—The study does not associate malocclusion to bottle or breast feeding.
Warren, J.J.; *et al.*; 2001 [205]	Not selected—The study does not report breast feeding or bottle feeding; only non-nutritive sucking habits.
Warren, J.J., *et al.*; 2005 [206]	Not selected—The study does not report breast feeding or bottle feeding; only non-nutritive sucking habits.
Watase, S., *et al.*; 1998 [207]	Not selected—The study is comprised of a high selected group of patients with otitis media. Other variables are associated to otitis media, but not malocclusion and feeding habits directly.
Westling, L., *et al.*; 1996 [208]	Not selected—The study does not report breast feeding or bottle feeding.
Winter, G.B.; 2002 [209]	Not selected—Letter to the Editor.
Yamazaki, S.; *et al.*, 2013 [210]	Not selected—The study does not associate malocclusion to bottle or breast feeding.
Zawawi, K.H.; *et al.*, 2012 [211]	Not selected—The study does not associate malocclusion to bottle or breast feeding.
Zhang, Q., *et al.*; 2014 [212]	Not selected—The study does not associate malocclusion to bottle or breast feeding.
Zuanon, A.C.C.; *et al.*, 2000 [213]	Not selected—The study reports oral habits.

References

1. Adair, S.M.; Milano, M.; Dushku, J.C. Evaluation of the effects of orthodontic pacifiers on the primary dentitions of 24- to 59-month-old children: Preliminary study. *Pediatr. Dent.* **1992**, *14*, 13–18.
2. Adamiak, E. Prevalence of occlusal disorders in preschool children in rural areas in relation to various individual factors. *Czas. Stomatol.* **1981**, *34*, 551–555.
3. Ahangar Atashi, M.H.; Shahamfar, M. Long-term evaluation of clinical performance of direct-bonded brackets: An epidemiologic survey. *J. Contemp. Dent. Pract.* **2013**, *14*, 738–742.
4. Ahlgren, J. EMG studies of lip and cheek activity in sucking habits. *Swed. Dent. J.* **1995**, 19, 95–101.
5. Al-Bajjali, T.T.; Rajab, L.D. Traumatic dental injuries among 12-year-old Jordanian schoolchildren: An investigation on obesity and other risk factors. *BMC Oral Health* **2014**, *14*, doi:10.1186/1472-6831-14-101.
6. Alexander, L.G. The thumb, the child, and the dentition or ignore the thumb, the tongue is worse. *J. Mass. Dent. Soc.* **1981**, *30*, 148–146.
7. Alexander, S.; Hegde, S.; Sudha, P. Prevalence of malocclusion and periodontal status in Tibetan school children of Kushalnagar, Mysore district. *J. Indian. Soc Pedod. Prev. Dent.* **1997**, *15*, 114–117.
8. Almeida, S.P.T.M.A.; Paixão, R.F.; Vieira, G.O. Influence of milk-feeding type, suction and malocclusion habits: Systemic review of literature. *J. Bras. Odonton. Ortop. Facial.* **2005**, *10,* 275–289.

9. Antonini, A.; Marinelli, A.; Baroni, G.; Franchi, L.; Defraia, E. Class II malocclusion with maxillary protrusion from the deciduous through the mixed dentition: A longitudinal study. *Angle Orthod*. **2005**, *75*, 980–986.
10. Aravena, A.P.; Alvarez, J.F.; Roizen, G.S. Effect of bad sucking habit in the dentition of children. *Pediatr. Día* **1998**, *14*, 269–272.
11. Aznar, T.; Galan, A.F.; Marin, I.; Dominguez, A. Dental arch diameters and relationships to oral habits. *Angle Orthodont*. **2006**, *76*, 441–445.
12. Backlund, E. Facial growth, and the significance of oral habits, mouthbreathing and soft tissues for malocclusion. A study on children around the age of 10. *Acta Odontol. Scand.* **1963**, *21*, 9–139.
13. Baragona, P.M.; Cohen, H.V. Long-term orthopedic appliance therapy. *Dent. Clin. North Amer.* **1991**, *35*, 109–121.
14. Baume, L.J. The pattern of dental disease in French polynesia. *Int. Dent. J.* **1973**, *23*, 579–584.
15. Baume, L.J. Uniform methods for the epidemiologic assessment of malocclusion. Results obtained with the World Health Organization standard methods (1962 and 1971) in South Pacific populations. *Amer. J. Orthod.* **1974**, *66*, 251–272.
16. Bertoldi, P.M.; Felficio, C.M.; Matsumoto, M.A. Effect of the early intervention of oral habits on the development of dental occlusion. *Pro. Fono*. **2005**, *17*, 37–44.
17. Bertrand, F.R. The relationship of prolonged breast feeding to facial features. *Cent. Afr. J. Med.* **1968**, *14*, 226–227.
18. Bigenzahn, W.; Fischman, L.; Mayrhoferkrammel, U. Myofunctional therapy in patients with orofacial dysfunctions affecting speech. *Folia Phoniat.* **1992**, *44*, 238–244.
19. Boni, R.C.; Veiga, M.C.F.A.; Almeida, R.C. Comportment of anterior open bite, after, the retreat the habit of sucking. *J. Bras. Orthod. Ortop. Maxilar*. **1997**, *12*, 35–40.
20. Borghoff, M.J.; Mombelli, M.L.; Murakami, R.M.; Goldenberg, F.C.; Bommarito, S. Breast feeding and the relationship with oral habits and malocclusions on mixture denture. *Odonto*. **2005**, *26*, 95–104.
21. Bowden, B.D. The effects of digital and dummy sucking on arch widths, overbite, and overjet: A longitudinal study. *Aust. Dent. J.* **1966**, *11*, 396–404.
22. Caglar, E; Larsson, E.; Andersson, E.M.; Hauge, M.S.; Ogaard, B.; Bishara, S.; Warren, J.; Noda, T.; Dolci, G.S. Feeding, artificial sucking habits, and malocclusions in 3-year-old girls in different regions of the world. *J. Dent. Child*. **2005**, *72*, 25–30.
23. Callaghan, A.; Kendall, G.; Lock, C.; Mahony, A.; Payne, J.; Verrier, L. Association between pacifier use and breast-feeding, sudden infant death syndrome, infection and dental malocclusion (Provisional abstract). *Int. J. Evid.-Based Healthcare* **2005**, *3*, 147–167.
24. Calo, F.A. Physiology and pathology resulting from sucking. *Rev. Circ. Argent. Odontol.* **1968**, *31*, 23–26.
25. Carrascoza, K.C.; Possobon, R.F.; Tomita, L.M.; Moraes, A.B. Consequences of bottle-feeding to the oral facial development of initially breastfed children. *J. Pediatr*. **2006**, *82*, 395–397.
26. Carrero, D.H.; Valls, D.W., Arenas, D.G. Functions of the stomatognathic system and occlusopathics. *Acta Odontol. Venez.* **1988**, *26*, 41–47.

27. Castellani, G.; Bertele, G.P.; Zerman, N. Epidemiologic study in nursery schools of the Verona Commune on the occurrence of dental caries, malocclusion and bad habits that can influence the normal development of facial bone structure in children. *Minerva. Stomatol.* **1987**, *36*, 121–125.
28. Castelo, P.M.; Gaviao, M.B.; Pereira, L.J.; Bonjardim, L.R. Maximal bite force, facial morphology and sucking habits in young children with functional posterior crossbite. *J. Appl. Oral. Sci.* **2010**, *18*, 143–148.
29. Castillo, B. Pacifier use in early infancy in relation to breast feeding, sudden infant death syndrome and poor dental occlusion. *Enferm. Clin*. **2008**, *18*, 223–225.
30. Castro, L.A.; Modesto, A.; Vianna, R.; Soviero, V.L.M. Estudo transversal da evolução da dentição decídua: Forma dos arcos, sobressaliência e sobremordida. *Pesqui. Odontol. Bras.* **2002**, *16*, 367–373. (In Portuguese)
31. Charchut, S.W.; Allred, E.N.; Needleman, H.L. The effects of infant feeding patterns on the occlusion of the primary dentition. *J. Dent. Child*. **2003**, *70*, 197–203.
32. Chevitarese, A.B.; Della, V.D.; Moreira, T.C. Prevalence of malocclusion in 4–6 year old Brazilian children. *J. Clin. Pediatr. Dent*. **2002**, *27*, 81–85.
33. Costantino, A. Class II malocclusion and bottle feeding. *Prev. Assist. Dent*. **1986**, *12*, 19–22.
34. Courson, F. Dental facial orthopedics in young children. *Arch. Pediatr*. **2006**, *13*, 679–682.
35. Cozza, P.; Baccetti, T.; Franchi, L.; Mucedero, M.; Polimeni, A. Sucking habits and facial hyperdivergency as risk factors for anterior open bite in the mixed dentition. *Amer. J. Orthod. Dent. Orthor.* **2005**, *128*, 517–519.
36. Degano, M.P.; Degano, R.A. Breastfeeding and oral health. A primer for the dental practitioner. *N. Y. State Dent. J.* **1993**, *59*, 30–32.
37. Del Valle, L.M.L.V.; Singh, G.D.; Feliciano, N.; Machuca, M.C. Associations between a history of breast feeding, malocclusion and parafunctional habits in Puerto Rican children. *P. R. Health Sci. J.* **2006**, *25*, 31–34.
38. Dimberg, L.; Bondemark, L.; Soderfeldt, B.; Lennartsson, B. Prevalence of malocclusion traits and sucking habits among 3-year-old children. *Swed. Dent. J.* **2010**, *34*, 35–42.
39. Dimberg, L.; Lennartsson, B.; Soderfeldt, B.; Bondemark, L. Malocclusions in children at 3 and 7 years of age: A longitudinal study. *Eur. J. Orthod.* **2013**, *35*, 131–137.
40. Diouf, J.S.; Ngom, P.I.; Badiane, A.; Cisse, B.; Ndoye, C.; Diop-Ba, K.; Diagne, F. Influence of the mode of nutritive and non-nutritive sucking on the dimensions of primary dental arches. *Int. Orthod.* **2010**, *8*, 372–385.
41. Djaha, K.; Adiko, E.F.; Ly, R.; Egnankou. K.J.; Brou, E.; Vilasco, J. Harmful oral habits of African children. *Odontostomatol. Trop*. **1986**, *9*, 51–55.
42. Dolci, G.S.; Ferreira, E.J.B.; Mello, A.L.S.F. Relations between sucking habits and malocclusions. *J. Bras. Odonton. Ortop. Facial.* **2001**, *6*, 379–385.
43. Dos Santos, R.R.; Nayme, J.G.; Garbin, A.J.; Saliba, N.; Garbin, C.A.; Moimaz, S.A. Prevalence of malocclusion and related oral habits in 5- to 6-year-old children. *Oral Health. Prev. Dent.* **2012**, *10*, 311–318.
44. Duncan, K.; McNamara, C.; Ireland, A.J.; Sandy, J.R. Sucking habits in childhood and the effects on the primary dentition: Findings of the Avon longitudinal study of pregnancy and childhood. *Int. J. Paediatr. Dent*. **2008**, *18*, 178–188.

45. Joanna Briggs Institute. Early childhood pacifier use in relation to breastfeeding, SIDS, infection and dental malocclusion. *Nurs. Stand.* **2006**, *20*, 52–55.
46. Emmerich, A.; Fonseca, L.; Elias, A.M.; de Medeiros, U.V. The relationship between oral habits, oronasopharyngeal alterations, and malocclusion in preschool children in Vitoria, Espirito Santo, Brazil. *Cad. Saude Publ.* **2004**, *20*, 689–697.
47. Eskes, P.W. Aetiology of malocclusion of the teeth. *Arch. Dis. Child.* **1992**, *67*, doi:10.1136/adc.66.9.1011.
48. Fabac, E.; Legouvic, M.; Zupan, M. The linkage between breast feeding and the growth of the orofacial area. *Fortschr. Kieferorthop.* **1992**, *53*,187–191.
49. Farsi, N.M.; Salama, F.S. Sucking habits in Saudi children: prevalence, contributing factors and effects on the primary dentition. *Pediatr. Dent.* **1997**, *19*, 28–33.
50. Ferreira, S.H.; Ruschel, H.C.; De Bacco, G.; Ulian, J. Prevalence of anterior open bite in 0–5 year-old children attended at day nurseries in Bento Gonçalves. *J. Bras. Odontopediatr. Odontol. Bebe.* **2001**, *4*, 74–79.
51. Ferrer, L.; Varela, V. Effect of the suction-swallowing action on orofacial development and growth. *Rev. Fac. Cien. Med. Univ. Nac. Cordoba.* **2006**, *63*, 33–37.
52. Ferro, A.; Cefariello, S. Prevention of malocclusion. *Arch. Stomatol.* **1979**, *20*, 387–410.
53. Fote, F.D.; Bosco, V.L. Prevalence of habits of non nutritive suction in Florianópolis-SC's children. *Rev. Fac. Odontol. Univ. Fed. Bahia.* **2000**, *20*, 25–28.
54. Fourquet, L.; Gottle, M.; Bounoure, G. Finishing and detailing, stability and harmony. *Orthod. Fr.* **2014**, *85*, 93–125.
55. Frazao, P.; Narvai, P.C. Socio-environmental factors associated with dental occlusion in adolescents. *Amer. J. Orthod. Dentofacial. Orthop*. **2006**, *129*, 809–816.
56. Furtado, A.N.M.; Vedovello Filho, M. The outcomes of the breast-feeding period in the settlement of nonnutritive sucking habits and in the occurrence of malocclusion in primary dentition. *RGO (Porto Alegre)* **2007**, *55*, 337–341.
57. Galan-Gonzalez, A.F.; Aznar-Martin, T.; Cabrera-Dominguez, M.E.; Dominguez-Reyes, A. Do breastfeeding and bottle feeding influence occlusal parameters? *Breastfeed Med.* **2014**, *9*, 24–28.
58. Gallarreta, F.W.M.; Silva, A.M.T.; Toniolo, I.M.F. Duration of breastfeeding and use of the bottle related to pacifier sucking habit and dental occlusion. *JPB. Rev. Ibero-am. Odontopediatr. Odontol. Bebê.* **2004**, *7*, 552–558.
59. Gandini, P.; Schiavi, A.; Camassa, D.; Manuelli, M. Statistical survey of malocclusion in school age children. *Mondo. Ortod.* **1989**, *14*, 73–78.
60. Gariner, D. Abnormal muscle function and the dentition. *Bull. N. J. Soc. Dent. Child.* **1970**, *17*, 6–10.
61. Garliner, D. Facts that every mother should know when choosing an artificial nursing system for her child. *Int. J. Orthod.* **1984**, *22*, 18–20.
62. Gedicke, K. On the predevelopment of the mandible in breast- and bottle-fed children from the standpoint of its function and the prevention of later distalization. *Monatsschr. Kinderheilkd.* **1961**, *109*, 361–369.

63. Gimenez, C.M.M.; Moraes, A.B.A.; Ambrosano, G.; Castro, F.M. First childhood malocclusion's prevalence and its correlation with breast feeding and oral habits. *J. Dent. Res.* **2001**, *80*, 1027–1033.
64. Gimenez, C.M.M.; Moraes, A.B.A.; Bertoz, A.D.; Bertoz, F.A.; Ambrosano, G.B. Prevalence of malocclusion in primary dentition and relation with feeding habits. *R. Dental. Press. Ortodon. Ortop. Facial.* **2008**, *13*, 70–83.
65. Gimenez, C.M.M.; Moraes, A.B.A.; Bertoz, A.P.; Bertoz, F.A.; Ambrosano, G.B. Prevalência de más oclusões na primeira infância e sua relação com as formas de aleitamento e hábitos infantis. *R. Dental. Press. Ortodon. Ortop. Facial.* **2008**, *13*, 70–83. (In Portuguese)
66. Gois, E.G.; Ribeiro-Junior, H.C.; Vale, M.P.; Paiva, S.M.; Serra-Negra, J.M.; Ramos-Jorge, M.L.; Pordeus, I.A. Influence of nonnutritive sucking habits, breathing pattern and adenoid size on the development of malocclusion. *Angle. Orthod.* **2008**, *78*, 647–654.
67. Grabowski, R.; Kundt, G.; Stahl, F. Interrelation between occlusal findings and orofacial myofunctional status in primary and mixed dentition: Part III: Interrelation between malocclusions and orofacial dysfunctions. *J. Orofac. Orthop.* **2007**, *68*, 462–476.
68. Gudin, R.G.; Khalef, M. Abnormalities in behavior in the oral and cervical area: reassurance breast-feeding. Considerations on sudden infant death. *Rev. Laryngol. Otol. Rhinol.* **1993**, *114*, 297–303.
69. Gyorgy, I. Prevention of occlusion anomalies. *Fogorv. Sz.* **1970**, *63*, 24–26.
70. Hannuksela, A.; Vaananen, A. Predisposing factors for malocclusion in 7-year-old children with special reference to atopic diseases. *Amer. J. Orthod. Dentofacial. Orthop.* **1987**, *92*, 299–303.
71. Harrel, S.K. More about occlusion. *J. Amer. Dent. Assn.* **2005**, *136*, 854–856.
72. Hashida, S.; Mihara, J.; Hashida, K.; Sumi, N.; Rakugi, M.; Ooshima, T.; Sobue, S. Clinical and statistical survey of the children at the Pedodontic Clinic of the Osaka University Dental Hospital. *Osaka. Daigaku. Shigaku. Zasshi.* **1985**, *30*, 336–344.
73. Hebling, S.R.; Cortellazzi, K.L.; Tagliaferro, E.P.; Hebling, E.; Ambrosano, G.M.; Meneghim, M.C.; Meneghim, M.C.; Pereira, A.C. Relationship between malocclusion and behavioral, demographic and socioeconomic variables: A cross-sectional study of 5-year-olds. *J. Clin. Pediatr. Dent.* **2008**, *33*, 75–79.
74. Heimer, M.V.; Katz, C.R.; Rosenblatt, A. Anterior open bite: A case-control study. *Int. J. Paediatr. Dent.* **2010**, *20*, 59–64.
75. Heimer, M.V.; Tornisiello Katz, C.R.; Rosenblatt, A. Non-nutritive sucking habits, dental malocclusions, and facial morphology in Brazilian children: a longitudinal study. *Eur. J. Orthod.* **2008**, *30*, 580–585.
76. Helle, A.; Haavikko, K. Prevalence of earlier sucking habits revealed by anamnestic data and their consequences for occlusion at the age of eleven. *Proc. Finn. Dent. Soc.* **1974**, *70*, 191–196.
77. Holberg, C.; Rudzki-Janson, I.; Wichelhaus, A.; Winterhalder, P. Ceramic inlays: Is the inlay thickness an important factor influencing the fracture risk? *J. Dent.* **2013**, *41*, 628–635.
78. Homan, B.T.; Davies, G.N. An oral health survey of Aborigines and Torres Strait Islanders in far North Queensland. *Aust. Dent. J.* **1973**, *18*, 75–87.
79. Ito, G. Skeletal and discrepancy factors in malocclusion. *Shikai. Tenbo.* **1984**, *63*, 1463–1470.

80. Iwashima, Y.; Kokubo, Y.; Ono, T.; Yoshimuta, Y.; Kida, M.; Kosaka, T.; Maeda, Y.; Kawano, Y.; Miyamoto, Y. Additive interaction of oral health disorders on risk of hypertension in a Japanese urban population: The Suita study. *Amer. J. Hypertens.* **2014**, *27*, 710–719.
81. Jabbar, N.S.; Bueno, A.B.; Silva, P.E.; Scavone-Junior, H.; Ines, F.R. Bottle feeding, increased overjet and Class 2 primary canine relationship: Is there any association? *Braz. Oral. Res.* **2011**, *25*, 331–337.
82. Jain, S.; Shetty, K.S.; Prakash, A.T.; Agrawal, M.; Jain, S. Permanent mandibular canine(s) impaction: Expansion of our understanding. *Aust. Orthod. J.* **2014**, *30*, 39–44.
83. Katz, C.R.; Rosenblatt, A.; Gondim, P.P. Nonnutritive sucking habits in Brazilian children: Effects on deciduous dentition and relationship with facial morphology. *Amer. J. Orthod. Dentofacial. Orthop.* **2004**, *126*, 53–57.
84. Katz, C.R.; Rosenblatt, A. Nonnutritive sucking habits and anterior open bite in Brazilian children: A longitudinal study. *Pediatr. Dent.* **2005**, *27*, 369–373.
85. Katz, C.R.T. Relationship among nonnutritive sucking habits, anterior open bite, posterior crossbite and facial morphology in children preschool in the city of Recife/PE, Brazil: A longitudinal study. Ph.D. Thesis, Department of Pediatric Dentistry, University of Pernambuco, Pernambuco, Brazil, 2003.
86. Kieser, J.J. Occlusal misconceptions. *Brit. Dent. J.* **2002**, *192*, doi:10.1038/sj.bdj.4801331a.
87. Kimmel, K. "With growth" nipple size—Important aid to jaw development. *Quintessenz. J.* **1990**, *20*, 1013–1017.
88. Kimmel, K. What became of them? I. A bottle nipple and a jaw-shaper, 25 years after their invention. *ZWR* **1980**, *89*, 62–67.
89. Kisling, E.; Krebs, G. Patterns of occlusion in 3-year-old Danish children. *Community. Dent. Oral. Epidemiol.* **1976**, *4*, 152–159.
90. Kobayashi, H.M.; Scavone Jr, H.; Ferreira, R.I.; Garib, D.G. Relationship between breastfeeding duration and prevalence of posterior crossbite in the deciduous dentition. *Amer. J. Orthod. Dentofacial. Orthop.* **2010**, *137*, 54–58.
91. Kobayashi, H.M.; Scavone Junior, H.; Ferreira, R.I.; Garib, D.G. Relanshionshipbetween non-nutritive sucking habits and posterior crossbite in the deciduous dentition. *Ortodontia.* **2008**, *41*, 367–372.
92. Koev, Z.H. Significance of breast feeding and bottle feeding in the development of the dentition. *Nauchni. Tr. Nauchnoizsled. Stomatol. Inst.* **1969**, *12*,135–140.
93. Kuijpers-Jagtman, A.M. Effects of sucking habits on the dentofacial development. *Ned. Tijdschr. Tandheelkd.* **1989**, *96*, 256–258.
94. Labbok, M.H.; Hendershot, G.E. Does breast-feeding protect against malocclusion? An analysis of the 1981 child health supplement to the national health interview survey. *Amer. J. Prev. Med.* **1987**, *3*, 227–232.
95. Larsson, E.; Jarvheden, B. Dummy- and finger-sucking habits with special attention to their significance for facial growth and occlusion. 2. Background variables. *Sven. Tandlak. Tidskr.* **1971**, *64*, 781–788.
96. Larsson, E. Artificial sucking habits: Etiology, prevalence and effect on occlusion. *Int. J. Orofacial. Myol.* **1994**, *20*, 10–21.

97. Larsson, E. Dummy- and finger-sucking habits in 4-year-olds. *Sven. Tandlak. Tidskr.* **1975**, *68*,219–224.
98. Larsson, E. Dummy- and finger-sucking habits with special attention to their significance for facial growth and occlusion. 1. Incidence study. *Sven. Tandlak. Tidskr.* **1971**, *64*, 667–672.
99. Larsson, E. Dummy- and finger-sucking habits with special attention to their significance for facial growth and occlusion. 3. Weaning. *Sven. Tandlak. Tidskr.* **1972**, *65*, 1–5.
100. Larsson, E. Effect of dummy-sucking on the prevalence of posterior cross-bite in the permanent dentition. *Swed. Dent. J.* **1986**, *10*, 97–101.
101. Larsson, E. Orthodontic aspects on feeding of young children. 1. A comparison between Swedish and Norwegian-Sami children. *Swed. Dent. J.* **1998**, *22*, 117–121.
102. Larsson, E. Sucking, chewing, and feeding habits and the development of crossbite: A longitudinal study of girls from birth to 3 years of age. *Angle. Orthod.* **2001**, *71*, 116–119.
103. Larsson, E. The effect of dummy-sucking on the occlusion: A review. *Eur. J. Orthod.* **1986**, *8*, 127–130.
104. Larsson, E.F.; Dahlin, K.G. The prevalence and the etiology of the initial dummy- and finger-sucking habit. *Amer. J. Orthod.* **1985**, *87*, 432–435.
105. Legovic, M.; Ostric, L. The effects of feeding methods on the growth of the jaws in infants. *ASDC J. Dent. Child.* **1991**, *58*, 253–255.
106. Leighton, B.C. Symposium on aspects of the dental development of the child. 2. The early development of cross-bites. *Dent. Pract. Dent. Rec.* **1966**, *17*, 145–152.
107. Leite-Cavalcanti, A.; Medeiros-Bezerra, P.K.; Moura, C. Breast-feeding, bottle-feeding, sucking habits and malocclusion in Brazilian preschool children. *Rev. Salud Publ.* **2007**, *9*, 194–204.
108. Linder, A.; Modeer, T. Relation between sucking habits and dental characteristics in preschoolchildren with unilateral cross-bite. *Scand. J. Dent. Res.* **1989**, *97*, 278–283.
109. Luft, R. Preventive measures for the prevention of malocclusion in infants. *Quintessenz* **1977**, *28*, 81–88.
110. Luz, C.L.; Garib, D.G.; Arouca, R. Association between breastfeeding duration and mandibular retrusion: A cross-sectional study of children in the mixed dentition. *Amer. J. Orthod. Dentofacial. Orthop.* **2006**, *130*, 531–534.
111. Macena, M.C.; Katz, C.R.; Rosenblatt, A. Prevalence of a posterior crossbite and sucking habits in Brazilian children aged 18–59 months. *Eur. J. Orthod.* **2009**, *31*, 357–361.
112. Maciel, C.T.; Leite, I.C. Etiological aspects of anterior open bite and its implications to the oral functions. *Pro. Fono.* **2005**, *17*, 293–302.
113. Maggioni, A. Bad habits that cause oral malocclusions. *Riv. Odontostomatol. Implantoprotesi.* **1984**, *8*, 104–106.
114. Mahalski, P.A.; Stanton, W.R. The relationship between digit sucking and behaviour problems: A longitudinal study over 10 years. *J. Child. Psychol. Psychiat.* **1992**, *33*, 913–923.
115. Malagola, C.; Mandraffino, A.G.; De Paolis, M. Role of feeding methods in the etiology of malocclusions. *Mondo. Ortod.* **1986**, *11*, 39–44.
116. Martins, R.J.; Forte, F.D.S.; Garbin, C.A.S.; Saliba, N.A. Ralationship between non-nutritive sucking habits and anterior open bite. *J. Health Sci. Inst.* **2003**, *21*, 401–404.

117. Martori, E.; Ayuso-Montero, R.; Martinez-Gomis, J.; Vinas, M.; Peraire, M. Risk factors for denture-related oral mucosal lesions in a geriatric population. *J. Prosthet. Dent.* **2014**, *111*, 273–279.
118. Masaracchia, R. Mother's breast or pacifier? Original or substitute? *Kinderkrankenschwester* **2009**, *28*, 454–456.
119. Massler, M. Oral habits: Development and management. *J. Pedod.* **1983**, *7*, 109–119.
120. Melink, S.; Vagner, M.V.; Hocevar-Boltezar, I.; Ovsenik, M. Posterior crossbite in the deciduous dentition period, its relation with sucking habits, irregular orofacial functions, and otolaryngological findings. *Amer. J. Orthod. Dent. Orthop.* **2010**, *138*, 32–40.
121. Melsen, B.; Stensgaard, K.; Pedersen, J. Sucking habits and their influence on swallowing pattern and prevalence of malocclusion. *Eur. J. Orthod.* **1979**, *1*, 271–280.
122. Mendes, A.C.R.; Valença, A.M.G.; Lima, C.C.M. Association between breast-feed, non-nutritive habits and malocclusions among children between 3 and 5 years old. *Cien. Odontol. Bras.* **2008**, *11*, 67–75.
123. Meyers, A.; Hertzberg, J. Bottle-feeding and malocclusion: Is there an association? *Amer. J. Orthod. Dent. Orthop.* **1988**, *93*, 149–152.
124. Michelotti, A.; Farella, M.; Buonocore, G.; Pellegrino, G.; Piergentili, C.; Martina, R. Is unilateral posterior crossbite associated with leg length inequality? *Eur. J. Orthod.* **2007**, *29*, 622–626.
125. Milicic, A. Prevention of orthodontic anomalies in infants. *Zobozdrav. Vestn.* **1972**, *27*, 41–44.
126. Milnes, A.R. Breastfeeding duration may be related to lower prevalence for posterior crossbite in the deciduous dentition. *J. Evid. Based Dent. Pract.* **2011**, *11*, 67–68.
127. Montaldo, L.; Montaldo, P.; Cuccaro, P.; Caramico, N.; Minervini, G. Effects of feeding on non-nutritive sucking habits and implications on occlusion in mixed dentition. *Int. J. Paediatr. Dent.* **2011**, *21*, 68–73.
128. Moraes, E.S.; Lira, C.C.; Ely, M.R.; Thomaz, E.B.A.F.; Valença, A.M.G. Prevalência de mordidas aberta e cruzada na dentição decídua. *Rev. Bras. Ciênc. Saúde.* **2001**, *5*, 23–30. (In Portuguese)
129. Moss, J.P.; Picton, D.C. The problems of dental development among the children on a Greek island. *Dent. Pract. Dent. Rec.* **1968**, *18*, 442–448.
130. Motokawa, M.; Terao, A.; Kaku, M.; Kawata, T.; Gonzales, C.; Darendeliler, M.A.; Tanne, K. Open bite as a risk factor for orthodontic root resorption. *Eur. J. Orthod*. **2013**, *35*, 790–795.
131. Mustafa, M.; Sidebottom, A. Risk factors for intraoperative dislocation of the total temporomandibular joint replacement and its management. *Brit. J. Oral. Maxillofac. Surg.* **2014**, *52*, 190–192.
132. Myllarniemi, S. Oral and dental state in Helsinki preschool children. V. Oral habits and occlusion. *Proc. Finn. Dent. Soc.* **1973**, *69*, 157–163.
133. Narbutyte, I.; Narbutyte, A.; Linkeviciene, L. Relationship between breastfeeding, bottle-feeding and development of malocclusion. *Stomatologija* **2013**, *15*, 67–72.
134. Neiva, F.C.; Cattoni, D.M.; Ramos, J.L.; Issler, H. Early weaning: Implications to oral motor development. *J. Pediatr.* **2003**, *79*, 7–12.
135. Nowak, A.J. Feeding and dentofacial development. *J. Dent. Res.* **1991**, *70*, 159–160.
136. Nystrom, M. Occlusal changes in the deciduous dentition of a series of Finnish children. *Proc. Finn. Dent. Soc.* **1981**, *77*, 288–295.

137. Obraztsov, I. A clinico-statistical analysis of the risk factors for the occurrence of maxillodental anomalies in children. *Stomatologiia. (Mosk)* **1990**, *70*, 66–69.
138. Ogaard, B.; Larsson, E.; Lindsten, R. The effect of sucking habits, cohort, sex, intercanine arch widths, and breast or bottle feeding on posterior crossbite in Norwegian and Swedish 3-year-old children. *Amer. J. Orthod. Dent. Orthop.* **1994**, *106*, 161–166.
139. Oikawa, K. Malocclusion in maternal and child health. *Shikai Tenbo* **1984**, *63*, 1431–1440.
140. Oliveira, A.B.; Souza, F.P.; Chiappetta, A.L.M.L. Relação entre hábitos de sucção não-nutritiva, tipo de aleitamento e má oclusões em crianças com dentição decídua. *Rev. CEFAC.* **2006**, *8*, 352–359. (In Portuguese)
141. Mew, J.O.; Thilander, B.; Lennartsson, B. A study of children with unilateral posterior crossbite, treated and untreated, in the deciduous dentition. Occlusal and skeletal characteristics of significance in prediction the long-term outcome. *J. Orofac. Orthop.* **2002**, *63*, 371–383.
142. Onyeaso, C.O.; Isiekwe, M.C. Oral habits in the primary and mixed dentitions of some Nigerian children: A longitudinal study. *Oral. Health. Prev. Dent.* **2008**, *6*, 185–190.
143. Onyeaso, C.O.; Isiekwe, M.C. Occlusal changes from primary to mixed dentitions in Nigerian children. *Angle. Orthod.* **2008**, *78*, 64–69.
144. Onyeaso, C.O. Need for preventive/interceptive orthodontic treatment among 7–10-year-old children in Ibadan, Nigeria: an epidemiological survey. *Odontostomatol. Trop.* **2004**, *27*, 15–19.
145. Ovsenik, M.; Farcnik, F.M.; Korpar, M.; Verdenik, I. Follow-up study of functional and morphological malocclusion trait changes from 3 to 12 years of age. *Eur. J. Orthod.* **2007**, *29*, 523–529.
146. Ovsenik, M. Incorrect orofacial functions until 5 years of age and their association with posterior crossbite. *Amer. J. Orthod. Dentofacial. Orthop.* **2009**, *136*, 375–381.
147. Page, D.C. Breastfeeding is early functional jaw orthopedics (an introduction). *Funct. Orthod.* **2001**, *18*, 24–27.
148. Pal, A. Observations on the dynamics and etiology of vertical anomalies of the dentition. *Fogorv. Sz.* **1976**, *69*, 129–133.
149. Paunio, P.; Rautava, P.; Sillanpaa, M. The Finnish family competence study: The effects of living conditions on sucking habits in 3-year-old Finnish children and the association between these habits and dental occlusion. *Acta. Odontol. Scand.* **1993**, *51*, 23–29.
150. Paunio, P.; Rautava, P.; Sillanpaa, M.; Kaleva, O. Dental health habits of 3-year-old Finnish children. *Community. Dent. Oral. Epidemiol*. **1993**, *21*, 4–7.
151. Paunio, P.; Rautava, P.; Sillanpää, M. The Finnish Family Competence Study: the effects of living conditions on sucking habits in 3-year-old Finnish children and the association between these habits and dental occlusion. *Acta. Odontol. Scand*. **1993**, *51*, 23–29.
152. Peters, S. Influence of early nursing on jaw and facial growth. *Quintessenz. J.* **1988**, *18*, 865–869.
153. Piras, V.; Tuveri, F.; Dessi, C.; Pittau, R.; Origa, R.; Basile, R.; Muroni, P.P.; Pittau, A. Relation between hypogonadism and malocclusion in beta-thalassemia major patients: Analysis of 122 subjects. *Minerva. Stomatol.* **2003**, *52*, 241–246.
154. Popovich, F. The prevalence of sucking habit and its relationship to oral malformations. *Appl. Ther.* **1966**, *8*, 689–691.

155. Primozic, J.; Farcnik, F.; Ovsenik, M.; Primozic, J. A controlled study of the functional and morphological characteristics of malocclusion in prematurely born subjects with low birth weight. *Eur. J. Orthod.* **2014**, *36*, 114–120.
156. Radovic, K.; Ilic, J.; Roganovic, J.; Stojic, D.; Brkovic, B.; Pudar, G. Denture stomatitis and salivary vascular endothelial growth factor in immediate complete denture wearers with type 2 diabetes. *J. Prosthet. Dent.* **2014**, *111*, 373–379.
157. Rakosi, T. Prevention of malocclusions and preventive orthodontics. *SSO Schweiz. Monatsschr. Zahnheilkd.* **1969**, *79*, 1169–1188.
158. Rakosi, T. Significance of early infancy in the development of malocclusion. *Zahnarztl. Prax.* **1972**, *23*, 321–322.
159. Raymond, J.L.; Bacon, W. Influence of feeding method on maxillofacial development. *Orthod. Fr.* **2006**, *77*, 101–103.
160. Ribeiro, H.C., Jr. Influence of nutritive and non-nutritive sucking habits on primary occlusion of brazilian children: A case-control study. Influence of nutritive and non-nutritive sucking habits on primary occlusion of Brazilian children: A case-control study. Ph.D. Thesis, Department of Pediatric Dentistry, Federal University of Minas Gerais, Belo Horizonte, Brazil, 2005.
161. Robke, F.J. Effects of nursing bottle misuse on oral health. Prevalence of caries, tooth malalignments and malocclusions in North-German preschool children. *J. Orofac. Orthop.* **2008**, *69*, 5–19.
162. Roedig. J.J.; Phillips. B.A.; Morford, L.A.; Van Sickels. J.E.; Falcao-Alencar, G.; Fardo, D.W.; Hartsfield, J.K., Jr.; Ding, X.; Kluemper, G.T. Comparison of BMI, AHI, and apolipoprotein E epsilon4 (APOE-epsilon4) alleles among sleep apnea patients with different skeletal classifications. *J. Clin. Sleep. Med.* **2014**, *10*, 397–402.
163. Romero, C.C.; Scavone-Junior, H.; Garib, D.G.; Cotrim-Ferreira, F.A.; Ferreira, R.I. Breastfeeding and non-nutritive sucking patterns related to the prevalence of anterior open bite in primary dentition. *J. Appl. Oral. Sci.* **2011**, *19*, 161–168.
164. Saenz, R.B. Breast-feeding and infant oral health. *Amer. Fam. Physician.* **2000**, *62*, 506–507.
165. Salma, S. Dentition and dental health. *Nurs. J. India.* **2000**, *91*, 102–104.
166. Sanchez-Molins, M.; Grau, C.J.; Lischeid, G.C.; Ustrell Torrent, J.M. Comparative study of the craniofacial growth depending on the type of lactation received. *Eur. J. Paediatr. Dent.* **2010**, *11*, 87–92.
167. Sanger, R.G.; Bystrom, E.B. Breast feeding: Does it affect oral facial growth? *Dent. Hyg.* **1982**, *56*, 44–47.
168. Scavone, H.; Guimaraes, C.H.; Ferreira, R.I.; Nahas, A.C.R.; Vellini-Ferreira, F. Association between breastfeeding duration and non-nutritive sucking habits. *Community Dent. Health* **2008**, 25, 161–165.
169. Scavone, H.; Ferreira, R.I.; Mendes, T.E.; Ferreira, F.V. Prevalence of posterior crossbite among pacifier users: a study in the deciduous dentition. *Braz. Oral. Res.* **2007**, *21*, 153–158.
170. Schneider, H.G.; Hierse, P.; Hierse, H.; Deichsel, E. Effect of the parental home on the oral health status of children. *Z. Gesamte. Hyg.* **1989**, *35*, 523–526.

171. Shekar, B.R.; Suma, S.; Kumar. S.; Sukhabogi, J.R.; Manjunath, B.C. Malocclusion status among 15 years old adolescents in relation to fluoride concentration and area of residence. *Indian. J. Dent. Res.* **2013**, *24*, 1–7.
172. Shendurnikar, N. Thumb sucking: practitioners' guidelines. J. Indian. Med. Assn. 1993, *91*. Available online: https://www.docphin.com/research/article-detail/12361473/PubMedID-8491980/Thumb-sucking-practitioners%27-guidelines (accessed on 12 March 2015).
173. Shetty, S.R.; Munshi, A.K. Oral habits in children-a prevalence study. *J. Indian Soc. Pedod. Prev. Dent*. **1998**, *16*, 61–66.
174. Silva, L.P.M.; Souza Júnior, M.A.; Bastos, E.P.S. Posterior cross-bite in deciduous dentintion. Relationship with oral habits. *Rev. Bras. Odontol.* **2005**, *62*, 49–51.
175. Simpson, W.J.; Cheung. D.K. Developing infant occlusion, related feeding methods and oral habits. Part I: Methodology and results at 4 and 8 months. *Dent. J.* **1976**, *42*, 124–132.
176. Simpson, W.J.; Cheung, D.K. Developing infant occlusion, related feeding methods and oral habits. Part II: Discussion and conclusions. *Dent. J.* **1976**, *42*, 135–137.
177. Solano, R.E.; Martin de Agar Valverde, M.C.; Mendoza, M.A. Study of etiopathogenic factors in Class II malocclusion. *Av Odontoestomatol.* **1988**, *4*, 69–79.
178. Sorensen, H.B.; Artmann, L.; Larsen, H.J.; Kjaer, I. Radiographic assessment of dental anomalies in patients with ectopic maxillary canines. *Int. J. Paediatr. Dent.* **2009**, *19*, 108–114.
179. Sousa, J.M.; Fracasso, M.L.C. Maternal behavior versus child temper: influence on the oral health pattern. Comportamento materno versus temperamento da criança: Influência no padrão de saúde bucal. *Pesq. Bras. Odontoped. Clín. Int*. **2010**, *10*, 47–54.
180. Souza Júnior, M.A. Contribution to the Study of the Posterior Cross-Bite Effect in Complete Primary Dentition. Ph.D. Thesis, Department of Pediatric Dentistry and Orthodontics, Federal University of Rio de Janeiro, Rio de Janeiro, Brazil, 1998.
181. Soviero, V.L.M. Study of the Influence of Feeding Methods and Durantion in the No Nutrition Sucking Habits Development and Dental Oclusion in the Primary Dentition. Ph.D. Thesis, Department of Pediatric Dentistry and Orthodontics, Federal University of Rio de Janeiro, Rio de Janeiro, Brazil, 1999.
182. Stahl, F.; Grabowski, R. Orthodontic findings in the deciduous and early mixed dentition—Inferences for a preventive strategy. *J. Orofac. Orthop.* **2003**, *64*, 401–416.
183. Starr, N.B.; Poland, C.; Dean, J.A. Malocclusion: How important is that bite? *J. Pediatr. Health Care* **1999**, *13*, 245–247.
184. Stecksen-Blicks, C.; Holm, A.K. Dental caries, tooth trauma, malocclusion, fluoride usage, toothbrushing and dietary habits in 4-year-old Swedish children: changes between 1967 and 1992. *Int. J. Paediatr. Dent.* **1995**, *5*, 143–148.
185. Stirling, D.L. Conditions contributing to stability and instability of dental alignment. *Ont. Dent.* **1994**, *71*, 48–50.
186. Taatz, H. Orthodontic prophylaxis. *Stomatol. DDR* **1978**, *28*, 342–351.
187. Takanashi, N.; Naya, K.; Masumori, M.; Nakano, J.; Ohama, A.; Hirata, J.; Akasaka, M. Survey on nutrition of infants in the Tokyo metropolitan area. 1. Carious dentitions, anomalies of occlusion and ways of eating. *Shoni Shikagaku Zasshi* **1989**, *27*, 708–715.

188. Thomaz, E.B.; Cangussu, M.C.; Assis, A.M. Malocclusion and deleterious oral habits among adolescents in a developing area in northeastern Brazil. *Braz. Oral. Res*. **2013**, *27*, 62–69.
189. Thomaz, E.B.; Cangussu, M.C.; Assis, A.M. Maternal breastfeeding, parafunctional oral habits and malocclusion in adolescents: A multivariate analysis. *Int. J. Pediatr. Otorhinolaryngol.* **2012**, *76*, 500–506.
190. Thomaz, E.B.A.F.; Valença, A.M.G. Prevalence of malocclusions and factors related to its occurence in preschool children in São Luís—MA—Brazil. *Rev. Pós-Graduação.* **2005**, *12*, 212–221.
191. Thomazine, G.D.P.A.; Imparato, J.C.P. Prevalência de mordida aberta e mordida cruzada em escolares da rede municipal de Campinas. *J. Bras. Odontopediatr. Odontol. Bebe.* **2000**, *3*, 29–37. (In Portuguese)
192. Tollaro, I.; Casini, R.G. Bad sucking habits and their repercussions on the structure of the face, mouth and jaws. *Riv. Clin. Pediatr.* **1968**, *81*, 1117–1124.
193. Tomita, L.M.; Carrascoza, K.C.; Possobon, R.F.; Ambrosano, G.M.B.; Moraes, A.B.A. Relação entre tempo de aleitamento materno, introdução de hábitos orais e ocorrência de maloclusões. *RFO UPF.* **2004**, *9*, 101–104. (In Portuguese)
194. Tomita, N.E.; Bijella, V.T.; Franco, L.J. The relationship between oral habits and malocclusion in preschool children. *Rev. Saude Publ.* **2000**, *34*, 299–303.
195. Tsai, H.H.; Sun, K.T. Growth changes of general and dental health status in Taiwanese children from mixed to early permanent dentition. *J. Clin. Pediatr. Dent.* **2004**, *28*, 309–314.
196. Tschill, P.; Bacon, W.; Sonko, A. Malocclusion in the deciduous dentition of Caucasian children. *Eur. J. Orthod.* **1997**, *19*, 361–367.
197. Turgeon-O'Brien, H.; Lachapelle, D.; Gagnon, P.F.; Larocque, I.; Maheu-Robert, L.F. Nutritive and nonnutritive sucking habits: A review. *J. Dent. Child.* **1996**, *63*, 321–327.
198. Urzal, V.; Braga, A.C.; Ferreira, A.P. The prevalence of anterior open bite in Portuguese children during deciduous and mixed dentition—Correlations for a prevention strategy. *Int. Orthod.* **2013**, *11*, 93–103.
199. Usadel, W. Breast feeding time and bite position. An analysis of 1000 anamneses of patients in an orthodontic practice. *Quintessenz* **1967**, *18*, 95–96.
200. Usadel, W. Duration of nursing and condition of bite. An evaluation of 1000 histories of patients from an orthodontics practice. *Quintessence. Int.* **1970**, *1*, 61–62.
201. Vasconcelos, F.M.; Massoni, A.C.; Heimer, M.V.; Ferreira, A.M.; Katz, C.R.; Rosenblatt, A. Non-nutritive sucking habits, anterior open bite and associated factors in Brazilian children aged 30–59 months. *Braz. Dent. J.* **2011**, *22*, 140–145.
202. Verrastro, A.P.; Stefani, F.M.; Rodrigues, C.R.; Wanderley, M.T. Occlusal and orofacial myofunctional evaluation in children with primary dentition, anterior open bite and pacifier sucking habit. *Int. J. Orofacial. Myol.* **2006**, *32*, 7–21.
203. Verrastro, A.P. Association between Nutritive and Non Nutritive Sucking Habits and Occlusal and Oral Myofunctional Characteristics in Children with Primary Dentition. Ph.D. Thesis, Department of Pediatric Dentistry, University of São Paulo, São Paulo, Brazil, 2008.
204. Vieira-Andrade, R.G.; Drumond, C.L.; Martins-Junior, P.A.; Correa-Faria, P.; Gonzaga, G.C.; Marques, L.S.; Ramos-Jorge, M.L.. Prevalence of sleep bruxism and associated factors in preschool children. *Pediatr. Dent.* **2014**, *36*, 46–50.

205. Warren, J.J.; Bishara, S.E.; Steinbock, K.L.; Yonezu, T.; Nowak, A.J. Effects of oral habits' duration on dental characteristics in the primary dentition. *J. Amer. Dent. Assn.* **2001**, *132*, 1685–1693.
206. Warren, J.J.; Slayton, R.L.; Bishara, S.E.; Levy, S.M.; Yonezu, T.; Kanellis, M.J. Effects of nonnutritive sucking habits on occlusal characteristics in the mixed dentition. *Pediatr. Dent.* **2005**, *27*, 445–450.
207. Watase, S.; Mourino, A.P.; Tipton, G.A. An analysis of malocclusion in children with otitis media. *Pediatr. Dent.* **1998**, *20*, 327–330.
208. Westling, L.; Mohlin, B. Palatal dimensions and some inherited factors (body height and metacarpal index). *Swed. Dent. J.* **1996**, *20*, 141–149.
209. Winter, G.B. Problems involved with the use of comforters. *Arch. Dis. Child.* **2002**, *87*, doi:10.1136/adc.87.2.170.
210. Yamazaki, S.; Arakawa, H.; Maekawa, K.; Hara, E.S.; Noda, K.; Minakuchi, H.; Sonoyama, W.; Matsuka, Y.; Kuboki, T. Retrospective comparative ten-year study of cumulative survival rates of remaining teeth in large edentulism. treated with implant-supported fixed partial dentures or removable partial dentures. *J. Prosthodont. Res.* **2013**, *57*, 156–161.
211. Zawawi, K.H.; Al-Harthi, S.M.; Al-Zahrani, M.S. Prevalence of gingival biotype and its relationship to dental malocclusion. *Saudi. Med. J.* **2012**, *33*, 671–675.
212. Zhang, Q.; Witter, D.J.; Bronkhorst, E.M.; Bartlett, D.W.; Creugers, N.H. Occlusal tooth wear in Chinese adults with shortened dental arches. *J. Oral. Rehabil.* **2014**, *41*, 101–107.
213. Zuanon, A.C.C.; Oliveira, M.F.; Giro, E.M.A.; Maia, J.P. Relação entre hábito bucal e maloclusão na dentadura decídua. *J. Bras. Odontopediatr. Odontol. Bebe.* **2000**, *3*, 104–108. (In Portuguese)

© 2015 by the authors; licensee MDPI, Basel, Switzerland. This article is an open access article distributed under the terms and conditions of the Creative Commons Attribution license (http://creativecommons.org/licenses/by/4.0/).
